# Herbal placebo response in clinical trials on irritable bowel syndrome: a systematic review and meta-analysis

**DOI:** 10.3389/fphar.2024.1475366

**Published:** 2024-11-28

**Authors:** Kaiyue Huang, Mi Lv, Ting Zheng, Fengyun Wang, Xudong Tang, Lin Lv

**Affiliations:** ^1^ Institute of Digestive Diseases, Xiyuan Hospital of China Academy of Chinese Medical Sciences, Beijing, China; ^2^ Graduate school, China Academy of Chinese Medical Sciences, Beijing, China

**Keywords:** herbs, complementary treatment, placebo, irritable bowel syndrome, meta-analysis

## Abstract

**Aim of the study:**

To systematically evaluate the herbal placebo response in randomized controlled trials (RCTs) of herbal medicine on irritable bowel syndrome (IBS).

**Materials and methods:**

We searched for RCTs with herbal placebo groups for IBS in PubMed, EMBASE, the Cochrane Library, the China National Knowledge Infrastructure (CNKI), the Wan Fang database and Sinomed database from 31 January 1994 to November 2023, and the quality of the literature was evaluated by the Cochrane risk of bias assessment criteria. The primary outcome indicators were response rate, abdominal pain and stool improvement rate, which were analyzed by single-group rate meta-analysis. Secondary outcomes were analyzed in subgroups based on diagnostic criteria, duration of treatment, subtype, research locations, placebo form, and presence of herbal ingredients to look for factors affecting respond rate.

**Results:**

The study included 24 papers, involving a total of 2,596 patients. Of these, 1151 IBS patients were treated with the herbal placebo. The placebo response rate in IBS patients in the herbal placebo group was 37% (P < 0.01,I^2^ = 75%). A total of 287 patients in five studies were given the herbal placebo, and the improvement rate of abdominal pain was 29% (P = 0.83, I^2^ = 0%). Four studies enrolled a total of 212 patients with IBS who received herbal placebo, and the stool improvement rate was 46% (P = 0.02 < 0.05, I^2^ = 71%). The research locations and treatment duration were sources of heterogeneity (P < 0.05).

**Conclusion:**

There is a significant herbal placebo response in patients with IBS. Different research locations and treatment durations are major sources of heterogeneity that may affect IBS patient response rates. The addition of a low dose of herbal ingredients when simulating an herbal placebo does not exaggerate the therapeutic effect of the placebo. There is a lack of uniformity and standardization in the preparation and evaluation of herbal placebos.

## Graphical Abstract

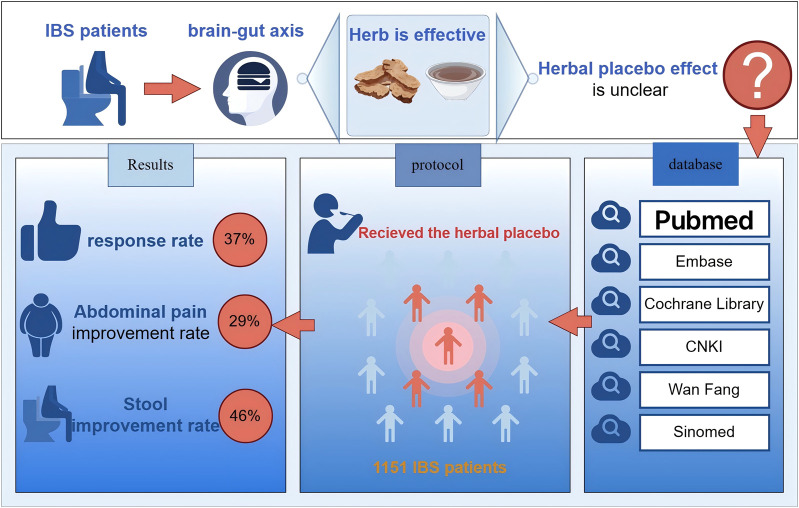

## 1 Introduction

In the past, irritable bowel syndrome (IBS) was thought to be a functional bowel disease that could not be explained by organic, structural abnormalities, but it is now widely recognized that IBS is mediated by brain-gut interaction disorders in functional gastrointestinal diseases (FGIDs) (Huang et al., 2023). Although the pathogenesis of IBS is not fully understood, it is now thought to be associated with genetics (e.g., SCN5A mutation), intestinal infections, microbial disorders, low-grade mucosal inflammation, immune activation, and bile acid metabolism disorders, and there is strong evidence for a brain-related connection (Holtmann et al., 2016). The prevalence of IBS varies significantly between locations, with the prevalence of IBS ranging from 1.1% (France and Iran) to 35.5% (Mexico), with large differences in prevalence between Europe and Asia (Black and Ford, 2020; Huang et al., 2023; Rahman et al., 2017; Sperber et al., 2017). Drugs recommended in the pharmacological treatment of IBS-D (diarrhea) include rifaximin to regulate intestinal flora, the mixed μ- and κ-opioid agonist/delta-opioid antagonist eludoline, and the selective 5-hydroxytryptamine 5-HT3 antagonist alosetron for the treatment of diarrhea and abdominal pain, but there are many limitations, such as unsatisfactory efficacy, strict indications, and adverse effects (Brenner and Sayuk, 2020; Nee et al., 2015). Due to the disappointment of conventional treatments, more and more people are looking for complementary and alternative medicine (CAM) treatments (Wu, 2010). Therefore, finding effective and safe CAM therapies is a critical issue that requires immediate attention nowadays. Herbal medicine, a multi-targeted therapy for the treatment of IBS, is a potential treatment for IBS by improving gastrointestinal motility, decreasing visceral hypersensitivity, and modulating intestinal flora (Chen et al., 2023; Shapiro and Deutsch, 2021). Herbs improved abdominal pain and global symptoms in patients with IBS compared to placebo (RR = 1.57, 95% CI: 1.31–1.88, I^2^ = 77%), but the quality of the current evidence is low and the lack of high-quality and rigorous RCTs of herbal medicines remains a challenge (Billings et al., 2021).

A high placebo response has been demonstrated in FGIDs with brain-gut axis interactions (Enck and Klosterhalfen, 2020), and IBS is one of the most common FGIDs associated with psychological effects, stress, and strain. One study showed that 27.3% of patients with IBS felt an overall improvement in symptoms after receiving placebo (Bosman et al., 2021). Therefore, in clinical RCTs, the IBS placebo response is important to control for the influence of non-treatment factors on the outcome; however, the herbal placebo response rate in IBS trials has not received attention. High-quality RCTs should account for the placebo effect. Therefore, we conducted a meta-analysis to clarify the herbal placebo response rate in patients with IBS in clinical trials to look for factors that may influence the placebo response rate and heterogeneity.

## 2 Materials and methods

This study was conducted in accordance with the updated 2020 Statement on Systematic Evaluation and Meta-Analysis. The protocol for this study is registered under PROSPERO registration number CRD42024509587, which can be found at https://www.crd.york.ac.uk/PROSPERO/display_record.php?RecordID=509587.

### 2.1 Search strategy

The English databases of PubMed, Embase, and Cochrane Library and the Chinese databases of CNKI, WanFang, and Sinomed were searched by two investigators combining the subject terms and free text words from January 1994 to November 2023. The terms of IBS: Irritable Bowel Syndrome and functional disease, colon [both as medical subject heading (MeSH) and free text terms] and Irritable Bowel Syndromes, Syndrome, Irritable Bowel, Syndromes, Irritable Bowel, Colon, Irritable, Irritable Colon, Colitis, Mucous, Colitides, Mucous, Mucous Colitides, Mucous Colitis, IBS, spastic colon (as free text terms). We combine these using the set operator AND with term identifiers of herbal and randomized controlled trials: Traditional Chinese Medicine, Drugs, Chinese Herba, Phytotherapy (both as MeSH and free text terms), and chinese medic*, chinese herb*, chinese drug*, chinese formul*, chinese plant, chinese prescri*, complementary therap*, alternativ* treatment*, alternativ* therap*, alternativ* medicin*, complementary therap* (as free text terms). And randomized controlled trial (publication type), randomized, placebo (as free text terms). And detailed search strategies for other databases are shown in Supplement. With the help of Endnote X9 to screen for duplicate literature, two researchers (H.K.Y. and Z.T.) independently read and evaluated the abstracts for literature screening, and when disagreements were encountered, L.L. participated in discussion and problem solving.

### 2.2 Inclusion criteria

We included the literature of clinical studies with herbal placebo control groups for the treatment of IBS, applying the following specific inclusion criteria: 1) Participants in the study must be over 18 years old, diagnosed with IBS based on Rome Criteria I-IV, and undergo a gastroscopy examination to rule out other organic diseases before recruitment. 2) Type of study design: RCTs meeting the criteria for double-blind, RCTs with a placebo group; 3) Intervention group setup: administration of herbal placebo soup, granules, proprietary Chinese medicines, poultices, and other herbal placebos as a test group, not receiving other basic treatments for IBS, and herbal medicines as a test group, with a treatment duration of ≥4 weeks; 4) Outcome indicators: the results of the study must report the response rate of the placebo group: response rate = (significant + improvement)/total patient cases × 100%; the number of extracted placebo-responsive subjects; and the information can be obtained directly from the articles or by calculation; 5) Language: English and Chinese.

### 2.3 Exclusion criteria

1) Duplicate papers with the most complete information were included, and all other duplicates were excluded; 2) Dissertations in Chinese databases; 3) Conference papers in Chinese databases; 4) Patients with significant symptom overlap in the study population. Dissertations and conference papers from Chinese databases were excluded because they were not peer-reviewed and there may be hidden risks to the rigor of the data (Supplementary Table S2). Furthermore, we excluded overlapping functional dyspepsia and gastroesophageal reflux disease due to their potential of biasing the results.

### 2.4 Data extraction

The basic information of the articles, including the first author, year of publication, title of the paper, country, language, diagnostic criteria, subtype, sample size, form of placebo, age, duration of administration, etc., was extracted by H.K.Y. and L.M., respectively, and recorded in the Excel 2021 form. If the efficiency is given directly, it is extracted directly; if it is given separately as relieve, significantly effective, improve, or ineffective, the data of the first two are taken (significant efficiency); the patients who fall off after enrollment in the placebo group are counted as invalid. Since it belongs to the analysis of the rate of a single group, when there is a full analysis set (FAS) and a per protocol set (PPS), the choice of the FAS is the result. When disagreements arose with the data, two individuals reexamined the entire text and reached a consensus through discussion.

### 2.5 Quality and risk of bias evaluation

The risk of bias evaluation of the included RCTs was evaluated using the evaluation entries of the RCT risk of bias evaluation tool recommended by the Cochrane Collaboration (Harbord et al., 2006; Higgins et al., 2011) and was evaluated by two reviewers (Z.T. and H.K.Y.) according to the entries, respectively. If differences were encountered, both of them reread the full text and resolved the differences by reaching a consensus through discussion. Also, the methods of Egger’s and Begg’s test (Sterne et al., 2011) were used to determine whether there was publication bias based on the P-value (the calibration level was α < 0.05).

### 2.6 Statistical analysis

R 4.2.3 software and the metaprop package were used to analyze the single group rate of response rate, abdominal pain improvement rate, and stool improvement rate, and the test of heterogeneity of the results of the included studies was analyzed using the X^2^ test, which was combined with the I^2^ quantification to determine the magnitude of the heterogeneity, and the inverted variance was used in the weighting method. The effect target data were transformed using the arcsine square root transformation, which was independent of the P-value without the need for continuous calibration. Setting the significance threshold as P< 0.05. We performed subgroup analyses based on various diagnostic criteria, treatment duration, subtype, study site, placebo form, and presence of herbal components. We used I^2^ and the p-value of Q tests to assess heterogeneity, which was defined as significant if I^2^ ≥ 50% and the p-value of Q tests <0.1 (Higgins et al., 2003). We analyzed placebo response rates using a random-effects model if there was significant statistical heterogeneity between studies.

## 3 Results

### 3.1 Search results

**FIGURE 1:**
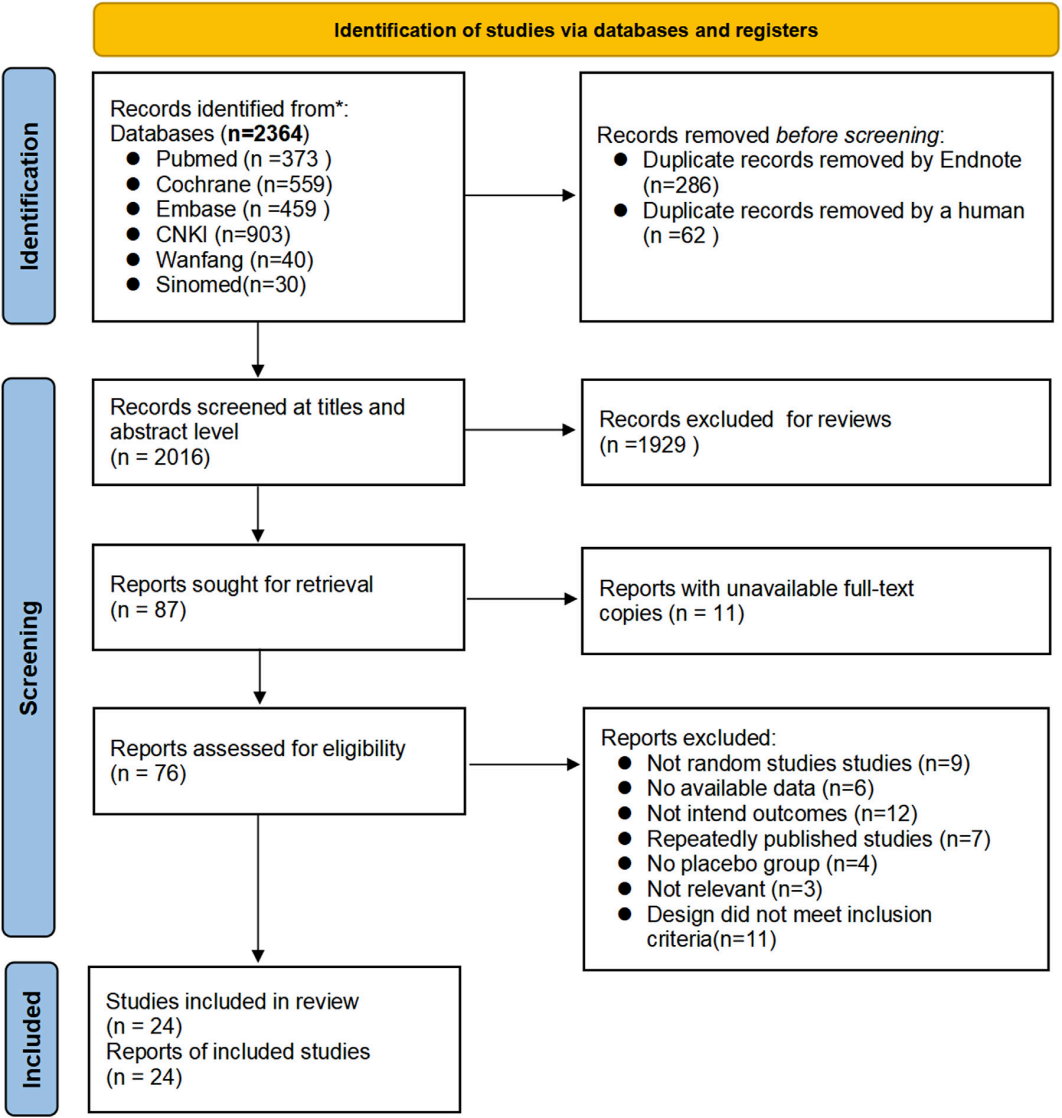
Literature screening flowchart.

Twenty-four articles (Alt et al., 2017; Bensoussan et al., 2015; Bensoussan et al., 1998; Cai et al., 2013; Chen et al., 2018; Davis et al., 2006; Heydari et al., 2023; Lai et al., 2022; Lee et al., 2019; Leung et al., 2006; Li et al., 2010; Madisch et al., 2004; Pazhouh et al., 2020; Portincasa et al., 2016; Saito et al., 2010; Sallon et al., 2002; Si et al., 2024; Størsrud et al., 2015; Su et al., 2013; Takeshi et al., 2014; Tang et al., 2018; Van Tilburg et al., 2014; Yu and Ma, 2011; Zhang et al., 2012) were finally screened to meet the inclusion criteria, including 19 English articles (Alt et al., 2017; Bensoussan et al., 2015; Bensoussan et al., 1998; Chen et al., 2018; Davis et al., 2006; Heydari et al., 2023; Lai et al., 2022; Lee et al., 2019; Leung et al., 2006; Madisch et al., 2004; Pazhouh et al., 2020; Portincasa et al., 2016; Saito et al., 2010; Sallon et al., 2002; Størsrud et al., 2015; Su et al., 2013; Takeshi et al., 2014; Tang et al., 2018; Van Tilburg et al., 2014) (79.17%) and 5 Chinese articles (Cai et al., 2013; Li et al., 2010; Si et al., 2024; Yu and Ma, 2011; Zhang et al., 2012) (20.83%), in which 1,151 patients with IBS were treated with herbal placebo, and the process of literature search is shown in Figure 1.

### 3.2 Basic characteristics

The basic characteristics of the 24 studies are shown in Table 1, including 12 (Alt et al., 2017; Bensoussan et al., 2015; Bensoussan et al., 1998; Chen et al., 2018; Davis et al., 2006; Lai et al., 2022; Madisch et al., 2004; Pazhouh et al., 2020; Portincasa et al., 2016; Si et al., 2024; Su et al., 2013; Tang et al., 2018) multicenter studies and 11 (Cai et al., 2013; Heydari et al., 2023; Lee et al., 2019; Leung et al., 2006; Li et al., 2010; Saito et al., 2010; Sallon et al., 2002; Takeshi et al., 2014; Van Tilburg et al., 2014; Yu and Ma, 2011; Zhang et al., 2012) single-center studies, 10 of which were conducted in China (Cai et al., 2013; Chen et al., 2018; Lai et al., 2022; Leung et al., 2006; Li et al., 2010; Si et al., 2024; Su et al., 2013; Tang et al., 2018; Yu and Ma, 2011; Zhang et al., 2012) and the rest in Australia, Europe, the Middle East, etc. (see Table 1 for details).

**TABLE 1:** The basic characteristics of 24 researches.

Trial	Location	Criteria/subtype	language	Study Population	Cases (E/P)	Age	Male/Female	treatment group	Duration frequency	form	Placebo response rate (%)
Alt et al. (2017)	European	RomeⅢ/IBS	English	Multicenter	43/47	47.5	32/47	IQP-CL-101, two capsule	8 weeks bid	capsule	27/47 (57%)
Bensoussan et al. (2015)	Australia	RomeⅢ/IBS-C	English	Multicenter	61/64	—	59/64	Bai Shao, Hou Pu, Zhi Ke, Chen Pi, Zhi Gan cao, Da Huang, Cang Zu	8 weeks bid	capsule	28/64 (44%)
Bensoussan et al. (1998)	Australia	RomeⅠ/IBS	English	Multicenter	38/43/35	45.0 ± 13.9	24/35	Individualized prescriptions	16 weeks tid	capsule	11/35 (31%)
Chen et al. (2018)	China	RomeⅢ/IBS-D	English	Multicenter	80/80	32.7 ± 8.2	49/80	Tong-Xie-Yao-Fang	4 weeks tid	granules	30/80 (38%)
Davis et al. (2006)	European	RomeⅡ/All	English	Multicenter	31/27	—	22/27	Aloe vera syrups, 50 mL	4 weeks qid	syrups	6/27 (22%)
Heydari et al. (2023)	Iranian	RomeⅢ/IBS-D	English	Single center	52/49	36 ± 7	20/38	75 mg of the dry extract of D. kotschyi and 175 mg of dibasic calcium phosphate as filler	4 weeks tid	capsule	8/49 (16%)
Lai et al. (2022)	China	RomeIV/IBS-D	English	Multicenter	120/120	40.9 ± 15.1	74/120	Addition and subtraction of Tong-Xie-Yao-Fang	4 weeks/N	liquid	42/120 (35%)
Lee et al. (2019)	Korea	RomeⅢ/IBS-D	English	Single center	20/20/20/20	45.2 ± 13.56	9/20	Samryungbaekchul-san	4 weeks tid	granules	7/20 (35%)
Leung et al. (2006)	China	RomeⅡ/IBS-D	English	Single center	60/59	43.6 ± 13.9	33/59	Addition and subtraction of Tong-Xie-Yao-Fang	8 weeks bid	granules	20/59 (34%)
Madisch et al. (2004)	European	RomeⅡ/All	English	Multicenter	51/52/53/52	46.1 ± 10.4	30/52	STW 5 and STW 5-II, 20 drops	4 weeks tid	liquid	20/52 (38%)
Pazhouh et al. (2020)	Iranian	RomeIV/IBS-C	English	Multicenter	35/35	34.89	25/35	formulated Persian herbal syrup, 15 mL	6 weeks tid	syrups	13/35 (37%)
Portincasa et al. (2016)	European	RomeⅢ/All	English	Multicenter	60/61	39.4	36/61	Curcumin and Fennel Essential Oil, two capsules	30 days bid	capsule	4/59 (7%)
Saito, et al. (2010)	America	RomeⅡ/All	English	Single center	35/35	42 (median)	30/35	St John’ s wort	12 weeks bid	tablet	21/35 (60%)
Sallon, et al. (2002)	Israeli	RomeⅠ/IBS-C	English	Single center	42/38	46.3 ± 2.9	28/38	Tibetan herbal formula, tow capsules	12 weeks qd	capsule	8/38 (21%)
Størsrud, et al. (2015)	European	RomeⅢ/All	English	—	33/35	44.2 ± 14.5	27/8	Aloe barbadensis Mill. Extract (AVH200^®^), 60 mg	4 weeks bid	tablet	11/35 (31%)
Su, et al. (2013)	China	RomeⅢ/IBS-D	English	Multicenter	120/120	37 ± 12	71/49	Modified Sishen Wan	4 weeks bid	tablet	53/120 (44%)
Takeshi, et al. (2014)	Japan	RomeⅢ/All	English	Single center	20/20	49.6 ± 16.0	9/20	Biobran, Modified Arabinoxylan Rice Bran	4 weeks bid	powder	6/20 (30%)
Tang et al. (2018)	China	RomeⅢ/IBS-D	English	Multicenter	109/107	42.4 ± 13.96	66/107	Chang’an I Recipe, 150 mL	8 weeks tid	liquid	43/107 (40%)
Van Tilburg et al. (2014)	America	RomeⅢ —	English	Single center	15/15/15	—	—	Ginger, 1 g/2g/day	4 weeks qd	capsule	8/15 (53%)
Cai et al. (2013)	China	RomeⅢ/IBS-D	Chinese	Single center	18/19	41.8 ± 9.33	4/14	decoction of dispersing stagnated liver-qi,invigorating spleen and warming kidney, 150 mL	8 weeks tid	liquid	11/19 (58%)
Li et al. (2010)	China	RomeⅢ/IBS-D	Chinese	Single center	30/30	36.6 ± 14.49	14/30	Chang Ji Tai Granule, 45 g/bag	4 weeks bid	granules	18/30 (60%)
Si et al. (2024)	China	RomeIV/IBS-D	Chinese	Multicenter	42/43/35	44.6 ± 13.40	14/35	Shuchang Decoction,12 g	8 weeks bid	granules	12/35 (34%)
Yu and Ma (2011)	China	RomeⅢ/All	Chinese	Single center	20/20/20	50.9 ± 8.93)	9/11	Emotional Intervention with Modified Pain-diarrhea Decoction, 100 mL	4 weeks tid	liquid	8/20 (40%)
Zhang, et al. (2012)	China	RomeⅢ/IBS-D	Chinese	Single center	42/30	—	—	Chang Ji Tai Granule	4 weeks bid	granules	16/30 (53%)

E, experimental group; P, placebo group; “/”, Lack of valid data. IBS-D, irritable bowel syndrome-diarrhea; IBS-C, irritable bowel syndrome-constipation; bid, bis in die (Latin, twice daily); tid, ter in die (Latin, three times a day); qid, quarter in die (Latin, four times a day); qd, quaque die (Latin, once a day).

### 3.3 Risk of bias assessment

**FIGURE 2:**
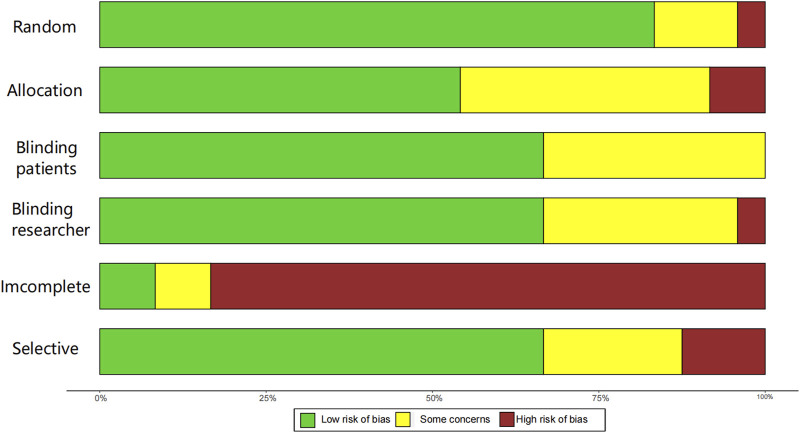
Assessment for risk of bias.

**FIGURE 3:**
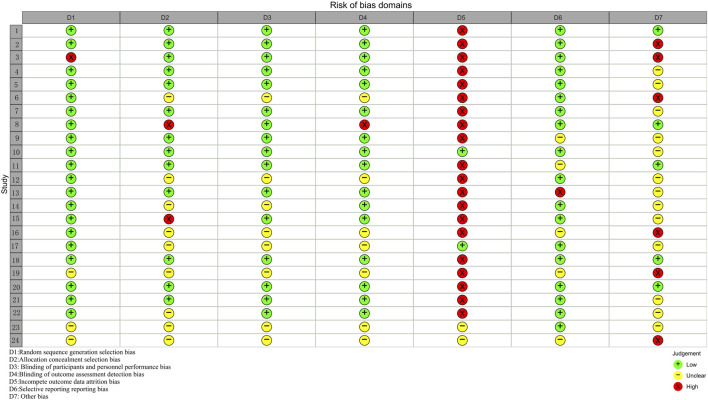
Risk of bias domains (Study 1–24 correspond in turn to references (Alt et al., 2017; Bensoussan et al., 2015; Bensoussan et al., 1998; Chen et al., 2018; Davis et al., 2006; Heydari et al., 2023; Lai et al., 2022; Lee et al., 2019; Leung et al., 2006; Madisch et al., 2004; Pazhouh et al., 2020; Portincasa et al., 2016; Saito et al., 2010; Sallon et al., 2002; Størsrud et al., 2015; Su et al., 2013; Takeshi et al., 2014; Tang et al., 2018; Van Tilburg et al., 2014; Cai et al., 2013; Li et al., 2010; Si et al., 2024; Yu and Ma, 2011; Zhang et al., 2012).

Twenty-four studies were evaluated using Cochrane’s RCT risk of bias, as described in the Supplement (Figures 2, 3; Supplementary Table S1). Three studies (Van Tilburg et al., 2014; Yu and Ma, 2011; Zhang et al., 2012) did not state the specific method of randomization. Nine studies (Alt et al., 2017; Heydari et al., 2023; Portincasa et al., 2016; Sallon et al., 2002; Si et al., 2024; Su et al., 2013; Takeshi et al., 2014; Van Tilburg et al., 2014; Yu and Ma, 2011; Zhang et al., 2012) did not state the method of allocating concealment. Seven studies (Heydari et al., 2023; Portincasa et al., 2016; Su et al., 2013; Takeshi et al., 2014; Van Tilburg et al., 2014; Yu and Ma, 2011; Zhang et al., 2012) did not state the specific recipients of the blinding method or the method of implementation, and one study (Lee et al., 2019) blinded patients only. The majority of studies had a fallout of the placebo group, and only two studies (Madisch et al., 2004; Takeshi et al., 2014) reported no placebo group fallout. The risk of bias was detected using R4.2.3 software, Begg’s test (P = 0.4534) and Egger’s test (P = 0.5157), and the P values were both > 0.05, indicating that there was no significant publication bias.

### 3.4 Placebo response rate

**FIGURE 4:**
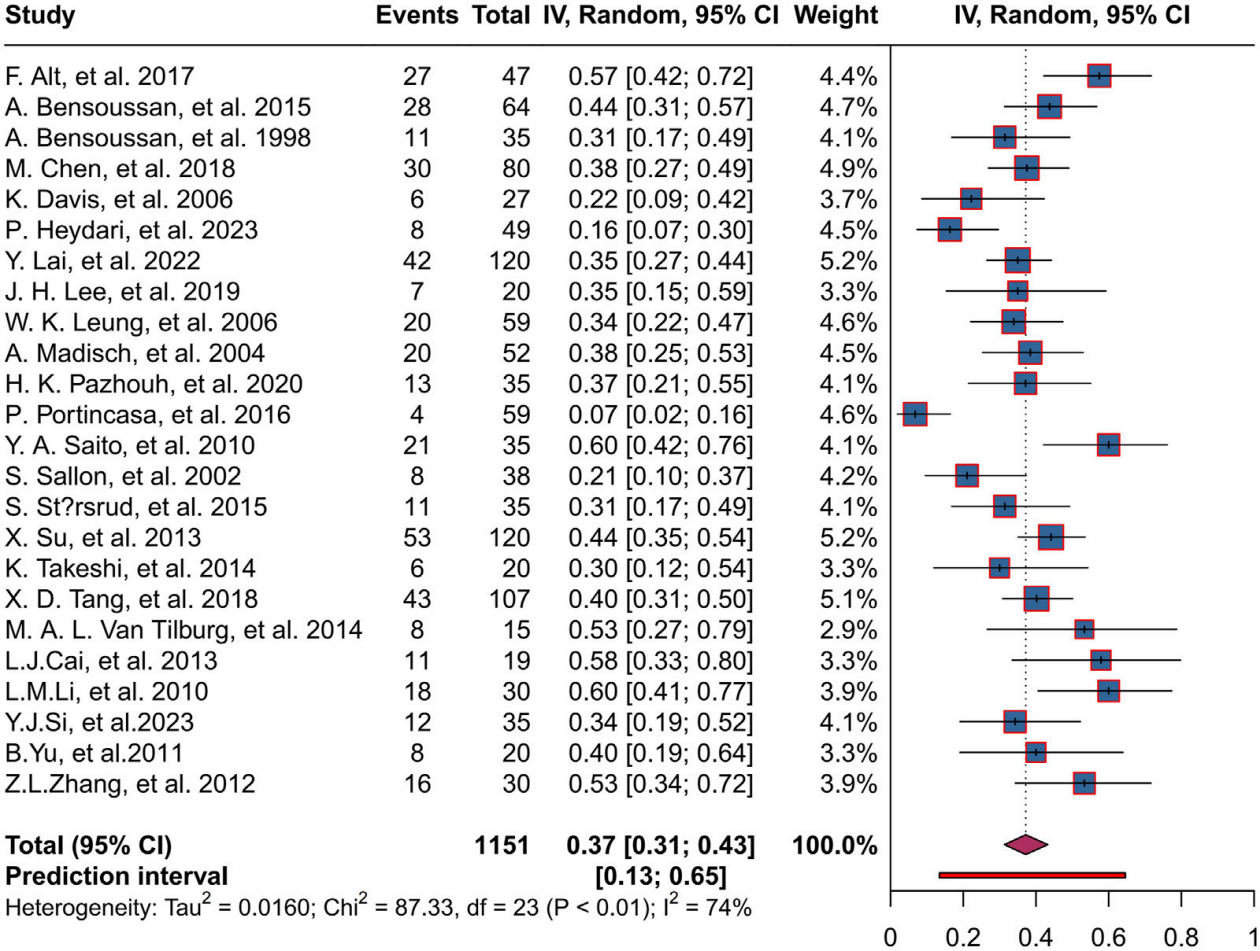
Forest plot of placebo response rate.

A total of 1,151 patients were enrolled in the placebo group in 24 trials, with a combined herbal placebo response rate of 37% (95% CI, 0.31 to 0.43; P < 0.01). There was significant heterogeneity between trials (I^2^ = 74%), using a random-effects model. The range of placebo response rates in the trials was 7%–60% (Figure 4).

### 3.5 Abdominal pain improvement rate

**FIGURE 5:**
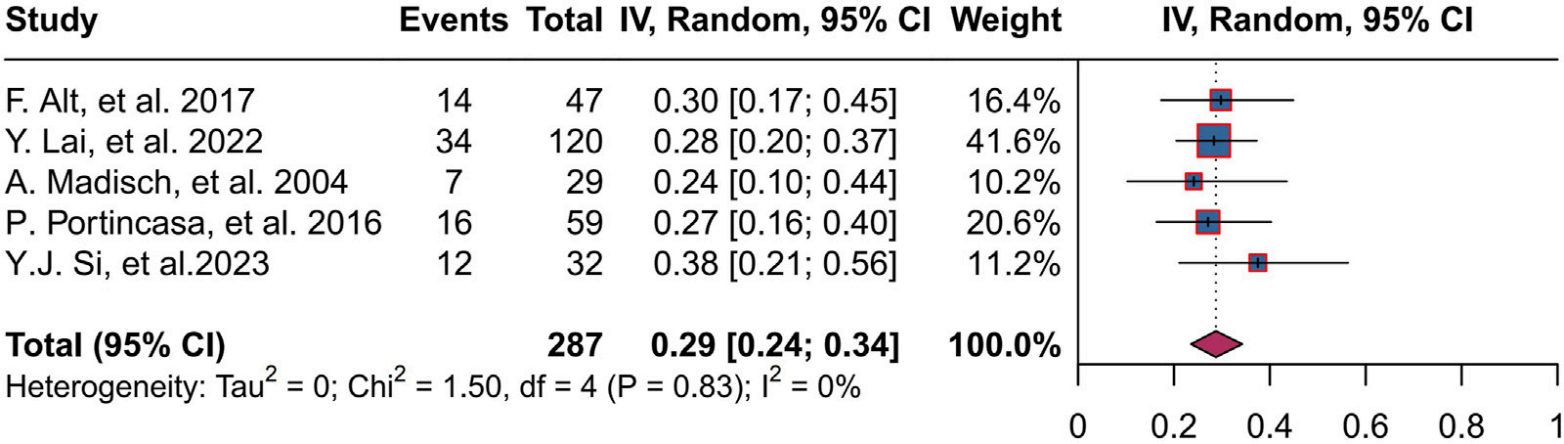
Forest plot of improved response rate for abdominal pain.

Five studies (Alt et al., 2017; Lai et al., 2022; Madisch et al., 2004; Portincasa et al., 2016; Si et al., 2024) reported an improvement rate in abdominal pain, totaling 287 patients, with a combined abdominal pain improvement rate of 29% (95% CI, 0.24 to 0.34; P = 0.83, I^2^ = 0%), and a range of abdominal pain improvement rates of 24%–38% across trials (Figure 5).

### 3.6 Stool improvement rate

**FIGURE 6:**
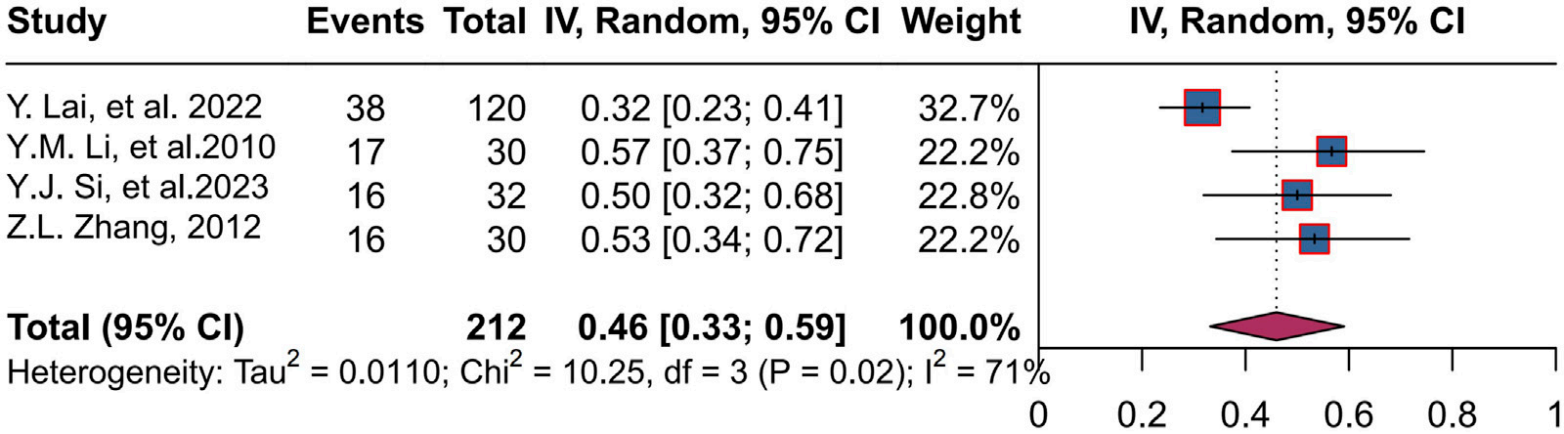
Forest plot of stool improvement response rate.

Four studies (Lai et al., 2022; Li et al., 2010; Si et al., 2024; Zhang et al., 2012) enrolled 212 patients, with a combined rate of 46% (95% CI, 0.33 to 0.59; P = 0.02 < 0.05, I^2^ = 71%), using a random-effects mode. The range of stool improvement rates across trials was 32%–57% (Figure 6).

### 3.7 Subgroup analysis

#### 3.7.1 Effect of diagnostic criteria on response rate

Subgroup analysis was conducted according to the different diagnostic criteria of Rome I-IV, among which 2 studies used Rome I diagnostic criteria, 4 studies used Rome II diagnostic criteria, 15 studies used Rome III diagnostic criteria, and 3 studies used Rome IV diagnostic criteria. The placebo response rates of Chinese medicine were 26%, 38%, 39%, and 35%, respectively (I^2^ = 0%, I^2^ = 70%, I^2^ = 80%, and I^2^ = 0%), and the difference between groups with different diagnostic criteria was not statistically significant (P = 0.28). For more information, please see Supplementary Figure S1.

#### 3.7.2 Effect of treatment duration on response rate

**FIGURE 7:**
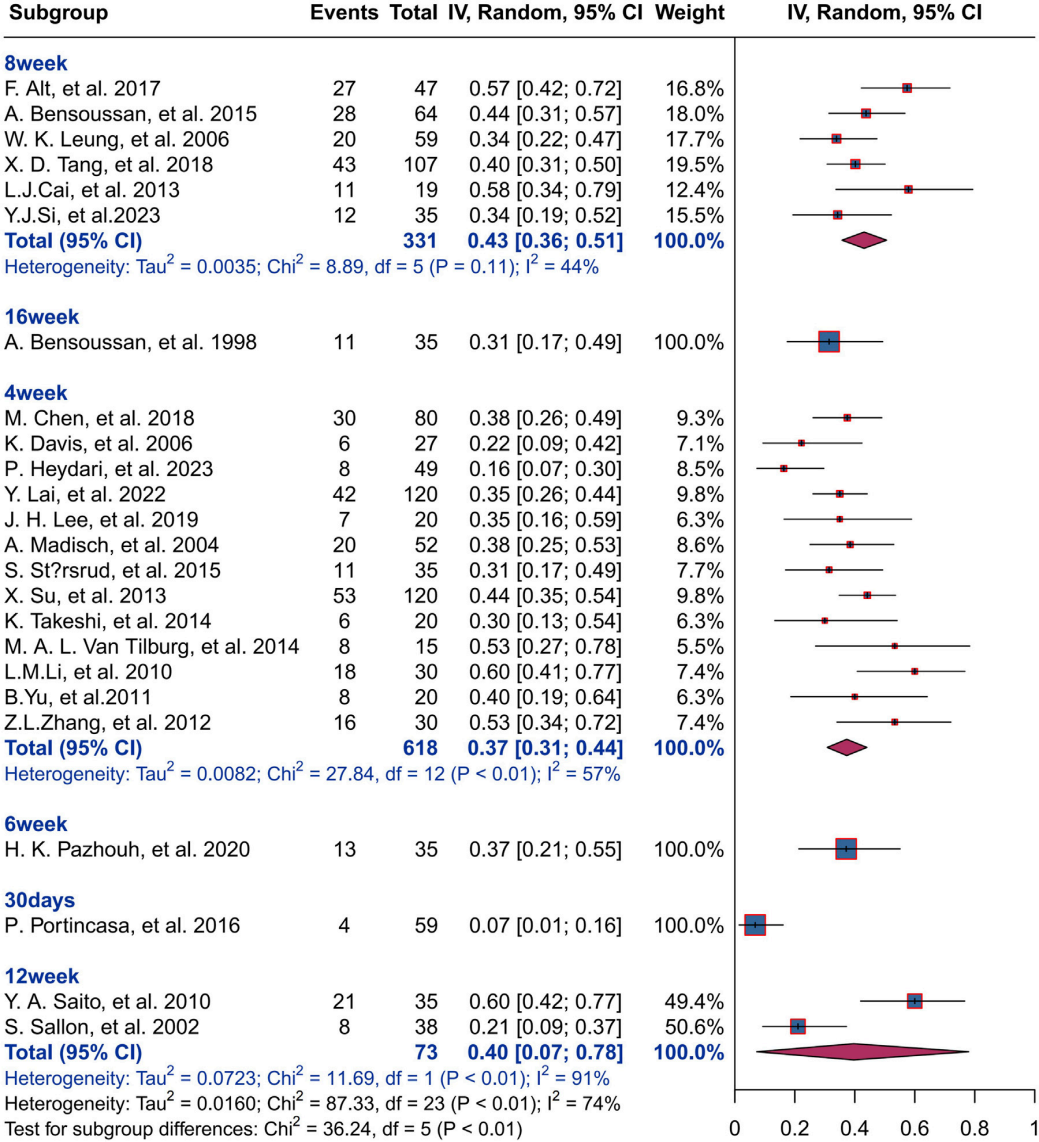
Effect of treatment duration on response rate.

The result of subgroup analysis based on treatment duration shows that 618 IBS patients in 13 studies were treated with herbal placebo for 4 weeks with a response rate of 37% (I^2^ = 57%, P< 0.01), and 331 IBS patients were treated for 8 weeks with a response rate of 43% (I^2^ = 44%, P< 0.01).73 IBS patients were treated for 12 weeks with a response rate of 40% (I^2^ = 91%, P< 0.01)There was statistically significant difference between groups for different treatment durations (P< 0.01). For more information, please see Figure 7.

#### 3.7.3 Effects of IBS subtypes on herbal placebo response rates

The funding of the subgroup analysis based on the IBS subtype revealed that in 11 studies including IBS-D subjects, the herbal placebo response rate was 39% in 669 IBS-D patients (I^2^ = 62%, P < 0.01) and a herbal placebo response rate of 34% in 137 IBS-C patients in 3 studies (I^2^ = 64%, P = 0.06). Nine studies did not differentiate between the subtypes of the study subjects. The response rate of IBS patients was 34% (I^2^ = 84%, P < 0.01). The difference between groups of different sybtypes was not statistically significant (P = 0.69). For more information, please see Supplementary Figure S2.

#### 3.7.4 Effects of research locations on herbal placebo response rates

**FIGURE 8:**
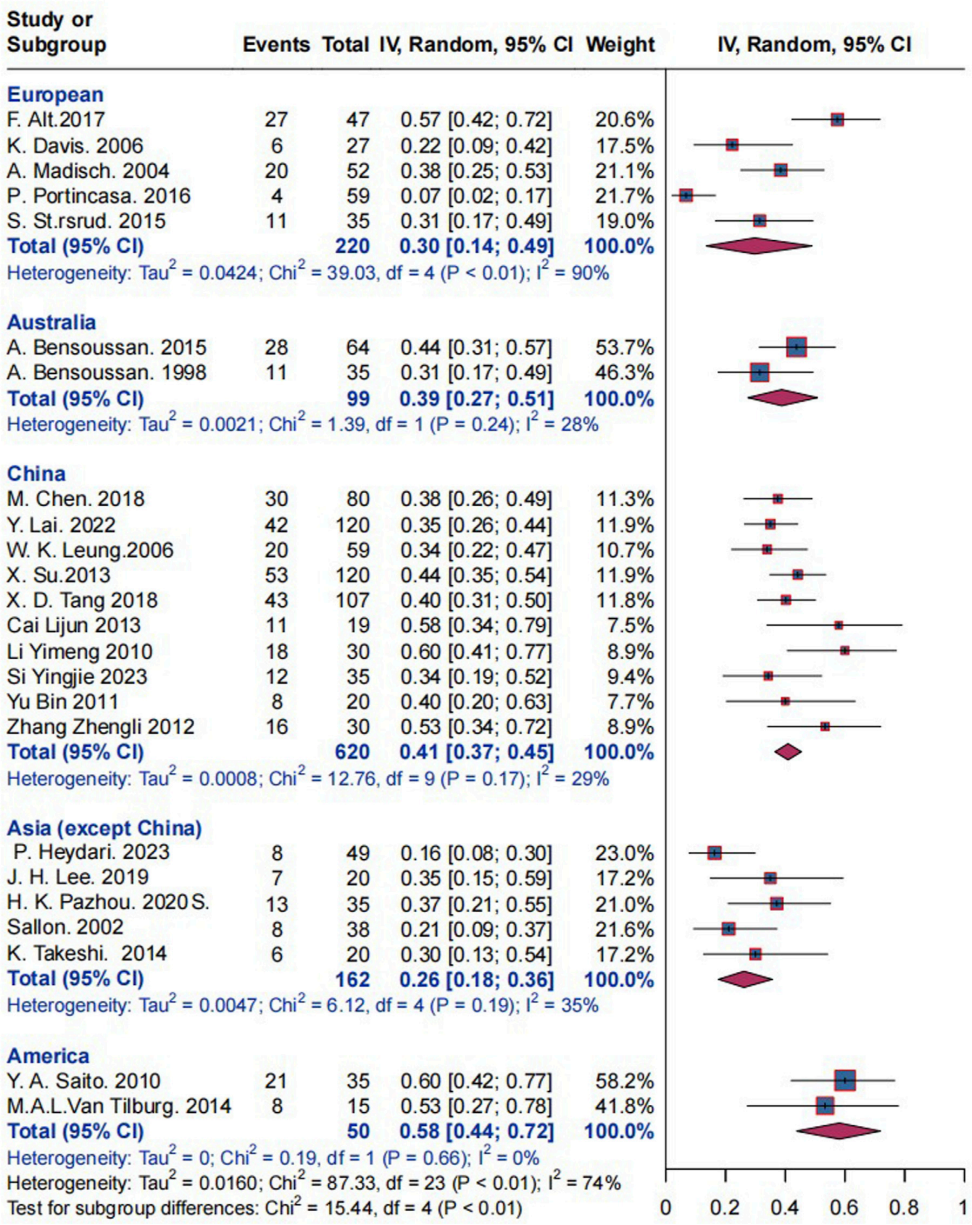
Effect of research locations on herbal placebo response rates.

Subgroup analysis based on research locations revealed that the placebo response rate in 220 European patients with IBS studies in five trials was 30% (I^2^ = 90%, P< 0.01), and the response rate in 99 Australian patients with IBS was 39% (I^2^ = 28%, P = 0.24). In 620 Chinese patients with IBS, the response rate was 41% (I^2^ = 29%, P = 0.17). The response for 162 Asian (excluding China) individuals with IBS was 26% (I^2^ = 35%, P = 0.19), while for 50 American IBS patients it was 58% (I^2^ = 0%, P = 0.17). Statistically significant differences were observed between the groups of the various research locations (P < 0.01); refer to Figure 8 for further information. China was analyzed separately and Asia was not included in the analysis because after many analyses, we found that there were significant differences in response rates while China was included in Asia (Supplementary Figure S6), and China was the country where herbal medicine was most commonly used, so we analyzed it independently.

#### 3.7.5 Effect of placebo forms on response rate

Seven studies used capsules with a placebo response rate of 31% (I^2^ = 88%, P< 0.01), five studies used granules with a response rate of 41% (I^2^ = 41%, P = 0.13), two studies used syrups with a response rate of 30% (I^2^ = 34%, P = 0.22), five studies used liquid with a response rate of 39% (I^2^ = 0%, P = 0.47), and three studies used tablets with a response rate of 45% (I^2^ = 65%, P = 0.06). There was no statistically significant difference between the groups (P = 0.5). Refer to the Supplementary Figure S3 for further information.

#### 3.7.6 Effect of low concentrations of herbal ingredients on response rate

According to the report in the results, four studies mentioned that the placebo ingredients contained low doses of the test group herbs, and 20 studies had placebos that did not contain the test group herbs. Subgroup analyses were performed based on this data, which showed that the response rate in the group containing the herbs was 46% (I^2^ = 50%, P = 0.11) and in the group not containing the herbs was 35% (I^2^ = 75%, P< 0.01), and the difference between the groups was not statistically significant (P = 0.12). See Supplementary Figure S4 for more details.

#### 3.7.7 Further analysis

Based on the results of subgroup analyses (Table 2), which revealed that the location of the study was a source of heterogeneity, we used it as a breakthrough point to further analyze the results. ① The research’s location of the study in and out of China: no difference between the two groups (P = 0.14). ② The language of the published article (Chinese and English), the difference between groups was statistically significant (P = 0.03 < 0.05), with 19 studies included in SCI journals published in English, with a response rate of 35% (I^2^ = 75%, P < 0.01). Five studies published in Chinese, with a response rate of 49% (I^2^ = 31%, P = 0.22). Overall, geography is a factor influencing herbal placebo response rates, especially in China, which is closely related to the historical development of herbal medicine in China. Returning to the study itself, it is reasonable that Chinese patients trust herbal medicines more, leading to a relatively higher response rate. See Supplementary Figure S6 for more details.

**TABLE 2:** The results of subgroup analysis.

Subtype	Number	Size	Proportion 95%CI	P-Value for I^2^
diagnostic criteria				
Rome I	2	73	0.26 (0.16; 0.37)	P = 0.32
Rome II	4	173	0.38 (0.25; 0.53)	P = 0.02
Rome Ⅲ	15	715	0.39 (0.30; 0.48)	P< 0.01
Rome Ⅳ	3	190	0.35 (0.28; 0.42)	P = 0.96
Duration of treatment				
4 weeks	13	618	0.37 (0.31; 0.44)	P< 0.01
8 weeks	6	331	0.43 (0.36; 0.51)	P = 0.11
12 weeks	2	73	0.40 (0.07; 0.78)	P< 0.01
IBS Subtypes				
IBS-C	3	137	0.34 (0.21; 0.48)	P = 0.06
IBS-D	11	669	0.39 (0.32; 0.46)	P< 0.01
All	9	330	0.34 (0.22; 0.47)	P< 0.01
research locations				
European	5	220	0.30 (0.14; 0.49)	P< 0.01
Australian	2	99	0.39 (0.27; 0.51)	P = 0.24
China	10	620	0.41 (0.37; 0.45)	P = 0.17
Asian (excluding China)	5	162	0.26 (0.18; 0.36)	P = 0.19
American	2	50	0.58 (0.44; 0.72)	P = 0.66
forms				
Capsules	7	307	0.31 (0.17; 0.47)	P< 0.01
granules	6	254	0.41 (0.33; 0.50)	P = 0.13
syrups	2	62	0.30 (0.17; 0.45)	P = 0.22
liquid	5	318	0.39 (0.33; 0.44)	P = 0.47
tablets	3	190	0.45 (0.31; 0.60)	P = 0.06
low concentration of herbal ingredients				
contained	4	202	0.46 (0.35; 0.56)	P< 0.01
Not contained	20	949	0.35 (0.29; 0.42)	P< 0.01
language				
English	19	1,017	0.35 (0.28; 0.41)	P< 0.01
Chinese	5	134	0.49 (0.38; 0.59)	P = 0.22

### 3.8 Meta-regression analysis

We conducted a meta-regression analysis of the subgroups using R 4.2.3 to identify the source of heterogeneity. We used criteria, duration of treatment, IBS subtype, research locations, placebo forms, and low concentration of herbal ingredients as covariates. We used a random effects model to determine whether *p* > 0.05 indicates that the factor is not a significant influence on heterogeneity, and *p* < 0.05 indicates that it is a source of heterogeneity. The results indicate that the location (*p* = 0.0438) and duration (*p* = 0.0365) may both be significant influencing factors for heterogeneity (see Supplementary Table S3 for an in-depth analysis).

### 3.9 Trial sequential analysis

**FIGURE 9:**
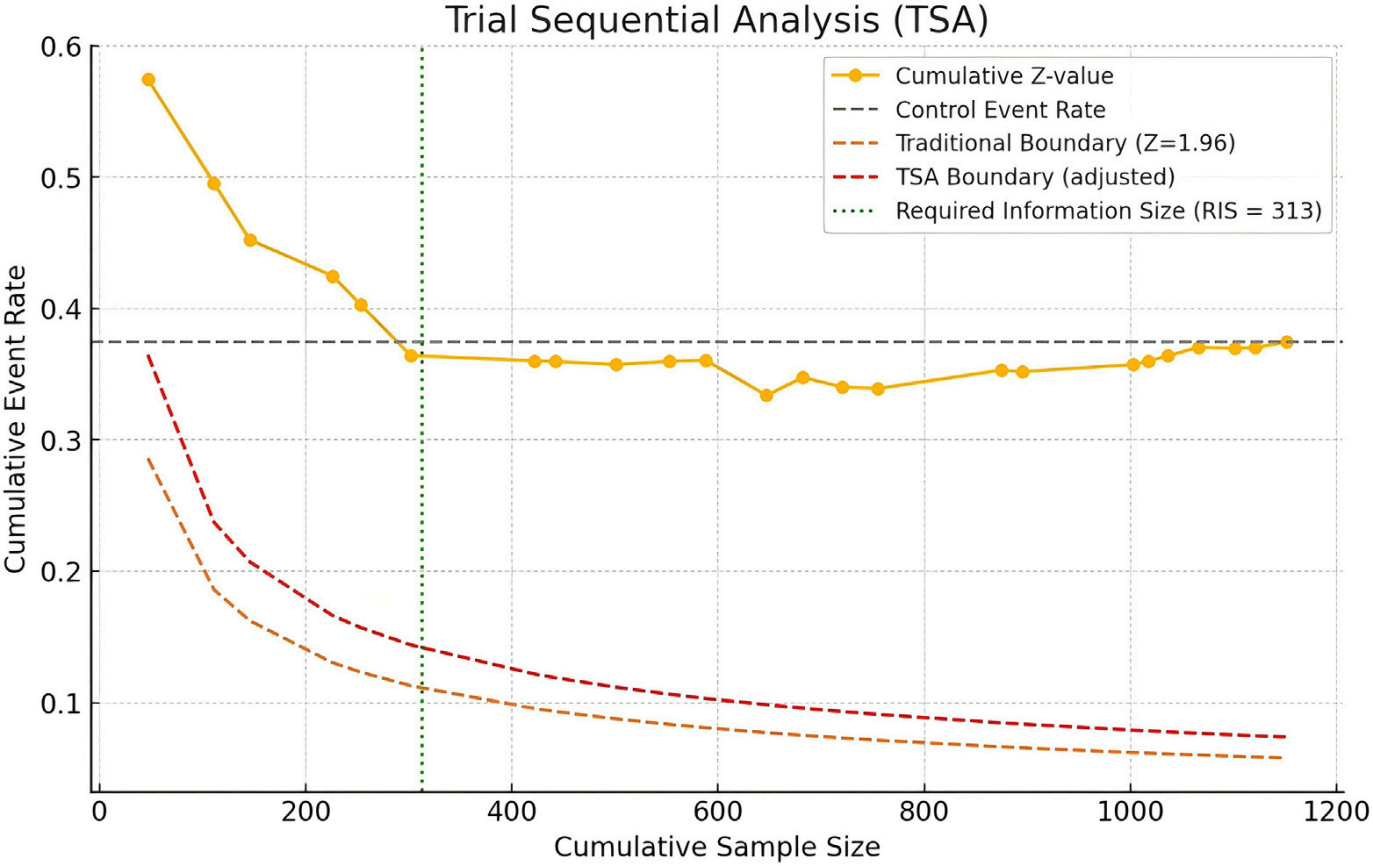
Trial sequential analysis for herbal placebo.

We used Python software to analyze the trial sequential analysis (TSA), allowing for a Type I error rate of 0.05, statistical efficacy of 80%. Z-curves did not cross the traditional boundaries, but whose cumulative information reaches the required information size (RIS), and the Z-curves has flattened out, indicating that there is no significant difference between the herbal placebo treatments and that this conclusion is also stable (Figure 9).

## 4 Discussion

Placebo is a tool that is frequently used in RCTs, and numerous studies in recent years have demonstrated significant placebo response rates for many diseases (Enck and Klosterhalfen, 2019; Jakovljevic, 2014); therefore, the placebo effect in patients should not be ignored in RCTs. Our study was the first to examine herbal placebos for IBS, and the results revealed a high herbal placebo response rate (37% in patients with IBS), with 29% reporting an improvement in abdominal pain and 49% reporting an improvement in bowel movements. Another study by Glissen Brown and Lembo (2022), showed that the combined composite response rate, abdominal pain rate, and fecal response rate for patients in the IBS-C placebo group, assessed at week 6, were 18.9%, 34.6%, and 30.1%, respectively. As per FDA standards, in the IBS-D placebo group, the combined placebo response rate for the composite endpoint was 16.2%, the abdominal pain improvement rate was 40.2%, and the fecal improvement rate was 16.2%. Bosman et al. (2021) included 73 RCTs of IBS in 2021, which showed an overall response rate of 34.4% and an improvement in abdominal pain of 17.9% in the placebo group for IBS. Comparing previous relevant studies, the placebo response rate in IBS patients varied considerably, and our findings are similar to the study by Bosman et al. Furthermore, our findings indicated that research locations and treatment durations were sources of heterogeneity in the placebo response rate and also affected the results of the herbal placebo response rate. The study by Bosman, M. et al. also found that the location of the study affected the placebo response rates in people with IBS. The pooled placebo response rate was significantly higher in trials conducted in Europe than in the United States (38.9% vs. 25.7%, *p* = 0.0032), but there was no significant difference in the pooled placebo response rate when compared to trials conducted in Asia (30.3%, *p* = 0.068). Our results also indicated that location plays a significant role in the effectiveness of herbal placebos in IBS patients, revealing a significantly higher herbal placebo response rate in China compared to other regions.

Because our study was focused on herbal medicines and China is a country where herbal medicines are more frequently used, we further analyzed the studies conducted in China and found that there was no difference in the herbal placebo response rate between China and countries outside of China (P = 0.14), but the difference in the herbal placebo response rate between the groups of studies where the language was English and Chinese was statistically significant (P = 0.03), so we believe that the design and rigor of the study is one of the reasons for the effect.

Based on the results of our study and the factors affecting the placebo effect, we will analyze it based on location, the patients themselves, and the placebo drug itself. In addition to this, placebo response rates are also related to the doctor-patient relationship, the recruitment process, and other external factors (Pardo-Cabello et al., 2022). To begin with, epidemiologic research on IBS has shown that the prevalence of IBS varies from country to country (Huang et al., 2023), and studies conducted globally by the Rome Foundation have shown comparable prevalence rates in Europe and the United States and slightly lower prevalence rates in Asia and Australia (Sperber, 2021). While there is no direct effect of prevalence and herbal placebo response rates, high prevalence and disease progression can affect efficacy as well as placebo response rates. The combination of IBS epidemiology and China is one of the most widely used countries for herbal medicine; the result is reliable and reasonable. Surprisingly, the IBS herbal placebo response rate in China was not the highest, and the herbal placebo response rate in the US was the highest in the two studies. As only two studies were conducted in the US, with only 50 participants, we suspect potential bias. In 10 studies in China, the herbal placebo response rate was as high as 41% (I^2^ = 29%) in 620 patients with IBS. We suspect potential bias. Given that China is the birthplace of Chinese herbal medicine and one of the countries with the widest use of herbal medicine, it is important to consider this high herbal placebo response rate when designing trials.

The second pertains to the placebo itself, including its visual, color, smell, dosage for, and pharmacological characteristics. The salience of the placebo plays a crucial role in determining the rates of placebo response (Buckalew and Coffield, 1982; Meissner and Linde, 2018). Currently herbal placebos lack uniform standards for production and evaluation, and there are challenges with odor and taste simulation, which can lead to low placebo response rates or even outright shedding if the placebo is easily recognized. So for the herbal placebo, we conducted subgroup analyses for the dosage form of the placebo and whether or not it contained an active ingredient, which also yielded reasonable and interesting results. Although there were no differences between the groups, the results showed that the highest response rates were found in the tablet and granule groups, and that granules are the most commonly used herbal placebo dosage form and the one that most closely resembles the original drug, which reduces patient recognition and reduces the error associated with placebo medications. The fact that tablets are less difficult to simulate in appearance is one of the reasons why they are less likely to be recognized. In addition to this, what is more interesting is that we found that the response rate of the placebo group made with added herbs was 35%, and the response rate of the placebo group made without herbs was 46%, which could also confirm that the addition of a small amount of herbs when making herbal placebos does not affect the outcome of the trial, and there are other relevant literature to prove this as well (Huang et al., 2024). Although there are fewer studies on the addition of herbs to make placebos, the results also proved that the addition of a small amount of the test group’s drug to the placebo does not cause the bias of placebo group’s response rate, and that the addition of a certain concentration of the original drug can make the placebo simulate the color, taste, and mouthfeel of the study drug better, which is a feasible option.

Both subgroup analyses and meta-regression analyses show that treatment duration does affect placebo response rates. We noticed that placebo response rates peaked at 8 weeks, with a positive correlation between medication duration and placebo response rates before 8 weeks and a negative correlation between medication duration and placebo response rates after 8 weeks. Expectation and learning are two of the most characterized primary mechanisms mediating the placebo response. At the beginning of the trial, patients may think that the treatment will alleviate their condition. This could lead to higher placebo response rates both at the beginning and as the trial goes on. However, as soon as the expectation is insufficient to mask the placebo’s inadequacy, the placebo response weakens.

Regarding the risk of bias, as the subject of this study was herbal placebos, and all studies set up a placebo group, so most of the trials achieved strict randomization, blinding, and allocation concealment. However, more than 80% (20/24) of the studies were found to have dropouts in the placebo group, and if the patients who dropped out were excluded from the study, the total number of participants in the study would have been reduced, and therefore would have inflated the placebo response rate. However, we believe that there is an inevitable link between patients who fall off and poor placebo efficacy, so we believe that all patients who fall off are due to poor efficacy, so our study categorizes patients who fall off as ineffective, so although most of the articles suffered from the bias of incomplete reporting, we tried to minimize the bias by not overstating the efficacy rate of the herbal placebo after treating the data in a uniform manner.

Placebo response in clinical trials is the result of a combination of psychological and neurobiological mechanisms, as well as natural history disease processes (Jones and Holtmann, 2023). Therefore, the herbal placebo response is influenced by numerous factors, especially the patient’s psychological feelings, and is a complex outcome. As countries with frequent use of herbal medicines, including China, Japan, Korea and so on. should pay more attention to the herbal placebo response when designing clinical trials involving herbal placebos. However, the search process could discover that many clinical trials of Chinese herbal medicines were not set up with standard blinding, even ignoring the herbal placebo effect. Therefore, setting up the placebo group is the key to improving the rigor and quality of clinical research on herbal medicines. In addition, our study found that adding a low level of herbal ingredients to the herbal placebo does not exaggerate the effect of the placebo and has no therapeutic effect on the placebo group. Herbal placebos are easily recognized by patients because of the difficulty in simulating the appearance, taste, and smell of herbal placebos. Adding a low level of the herbal ingredients can better simulate the herb placebo, which can solve the contradiction of the difficulty in simulating the placebo without setting a placebo group.

## 5 Limitations

1) Heterogeneity is high due to the different trial group prescriptions in each RCT and the uncontrollable baseline of patients taking herbal placebos. 2) Although most studies implemented blinding and allocation concealment, herbal placebos are difficult to simulate and so are easily recognized by patients, so the resulting outcomes are inevitably at risk of bias. In addition, the placebo group also shed a large number of patients, inevitably resulting in follow-up bias. 3) The criteria for evaluating the outcomes of the herbal RCTs are also not standardized, which would result in a risk of bias. 4) Herbal placebo response is related to the psychology of the patient, which varies between studies and can be deviated by the psychological status of the patient.

## 6 Conclusion

Primarily, there is a high herbal placebo response in IBS disease, where patient adherence and trust in the herbal medicine affects the outcome, and the placebo response should be emphasized in both trials and clinics. At the same time, we found that most Chinese RCTs of herbal medicines lacked placebo groups, and fewer studies met the inclusion criteria than English-language journals. Moreover, there was also a significant difference in the placebo response rates for the herbs summarized in the Chinese and English articles, indicating that the quality of the studies also affected the placebo response rates for the herbs. Finally, in light of the non-negligible placebo response rate for herbal medicines, we suggest that when conducting herbal medicine-related RCTs, if the disease or drug involved is ethical, there should be a placebo control group to exclude errors and to prove the validity and safety of the positive control drug, so that the trial design and results will be more rigorous and scientific. We should quantify and scientifically develop the production of herbal placebos and the standardized evaluation of appearance, taste, and odor to establish a standard and scientific evaluation system. This will reduce patient or researcher blindness, exclude the placebo response and confounding factors, and ensure the experimental group’s results are relatively reliable, rigorous, and scientific.

## Data Availability

The original contributions presented in the study are included in the article/Supplementary Material, further inquiries can be directed to the corresponding author.

## Abbreviations

RTCs, randomized controlled trial; IBS, irritable bowel syndrome; IBS-D, irritable bowel syndrome-diarrhea; IBS-C, irritable bowel syndrome-constipation; CNKI, China National Knowledge Infrastructure; WanFang, Wan Fang data knowledge service platform; SinoMed, China biomedicine literature database; FGIDs, functional gastrointestinal diseases; FAS, full analysis set; PPS, per protocol set.
