# Supplementary material for: Herbal placebo response in clinical trials on irritable bowel syndrome: a systematic review and meta-analysis
Source: Front Pharmacol. 2024 Nov 28;15:1475366. doi: 10.3389/fphar.2024.1475366 (PMC11634590; doi:10.3389/fphar.2024.1475366)
Supplement: Supplementary file 1 [file Table1.docx]

| Trial | Location | Criteria/subtype | language | Study Population | Cases  (E/P) | Age | Male/  Female | treatment group | Duration  frequency | form | Placebo response rate (%) |
| --- | --- | --- | --- | --- | --- | --- | --- | --- | --- | --- | --- |
| F. Alt,  et al.2017^[15]^ | European | RomeⅢ/  IBS | English | Multicenter | 43/**47** | 47.5 | 32/47 | IQP-CL-101, two capsule | 8week bid | capsule | 27/47(57%) |
| A. Bensoussan, et al.2015^[16]^ | Australia | RomeⅢ/  IBS-C | English | Multicenter | 61/**64** | / | 59/64 | Bai Shao、Hou Pu、Zhi Ke、Chen Pi、Zhi Gan cao、Da Huang、Cang Zu | 8week bid | capsule | 28/64(44%) |
| A. Bensoussan, et al.1998^[17]^ | Australia | RomeⅠ/  IBS | English | Multicenter | 38/43/  **35** | 45.0±13.9 | 24/35 | Individualized prescriptions | 16week tid | capsule | 11/35(31%) |
| M. Chen, et al.2018^[18]^ | China | RomeⅢ/  IBS-D | English | Multicenter | 80/**80** | 32.7± 8.2 | 49 /80 | Tong-Xie-Yao-Fang | 4week tid | granules | 30/80(38%) |
| K. Davis, et al.2006^[19]^ | European | RomeⅡ/  All | English | Multicenter | 31/**27** | / | 22/27 | Aloe vera syrups, 50ml | 4week qid | syrups | 6/27(22%) |
| P. Heydari, et al.2023^[20]^ | Iranian | RomeⅢ/  IBS-D | English | Single center | 52/**49** | 36 ±7 | 20/38 | 75 mg of the dry extract of D. kotschyi and 175 mg of dibasic calcium phosphate as filler | 4week tid | capsule | 8/49(16%) |
| Y. Lai, et al.2022^[21]^ | China | RomeIV/  IBS-D | English | Multicenter | 120/**120** | 40.9± 15.1 | 74/120 | Addition and subtraction of Tong-Xie-Yao-Fang | 4week /N | liquid | 42/120(35%) |
| J. H. Lee, et al.2019^[22]^ | Korea | RomeⅢ/  IBS-D | English | Single center | 20/20/  20/**20** | 45.2± 13.56 | 9 /20 | Samryungbaekchul-san | 4week tid | granules | 7/20(35%) |
| W. K. Leung, et al.2006^[23]^ | China | RomeⅡ/  IBS-D | English | Single center | 60/**59** | 43.6±13.9 | 33/59 | Addition and subtraction of Tong-Xie-Yao-Fang | 8week bid | granules | 20/59(34%) |
| A. Madisch, et al.2004^[24]^ | European | RomeⅡ/  All | English | Multicenter | 51/52/  53/**52** | 46.1±10.4 | 30/52 | STW 5 and STW 5-II, 20 drops | 4week tid | liquid | 20/52(38%) |
| H. K. Pazhouh, et al.2020^[25]^ | Iranian | RomeIV/  IBS-C | English | Multicenter | 35/**35** | 34.89 | 25/35 | formulated Persian herbal syrup, 15ml | 6week tid | syrups | 13/35(37%) |
| P. Portincasa, et al.2016^[26]^ | European | RomeⅢ/  All | English | Multicenter | 60/61 | 39.4 | 36/61 | Curcumin and Fennel Essential Oil, two capsules | 30days bid | capsule | 4/59(7%) |
| Y. A. Saito, et al.2010^[27]^ | America | RomeⅡ/  All | English | Single center | 35/**35** | 42(median) | 30/35 | St John ’ s wort | 12week bid | tablet | 21/35(60%) |
| S. Sallon, et al.2002^[28]^ | Israeli | RomeⅠ/  IBS-C | English | Single center | 42/38 | 46.3±2.9 | 28/38 | Tibetan herbal formula, tow capsules | 12week qd | capsule | 8/38(21%) |
| S. Størsrud, et al.2015^[29]^ | European | RomeⅢ/  All | English | / | 33/**35** | 44.2±14.5 | 27/8 | Aloe barbadensis Mill. Extract (AVH200®), 60mg | 4week bid | tablet | 11/35（31%） |
| X. Su, et al.2013^[30]^ | China | RomeⅢ/  IBS-D | English | Multicenter | 120/**120** | 37±12 | 71/49 | Modified Sishen Wan | 4week bid | tablet | 53/120(44%) |
| K. Takeshi, et al.2014^[31]^ | Japan | RomeⅢ/  All | English | Single center | 20/**20** | 49.6± 16.0 | 9/20 | Biobran, Modified Arabinoxylan Rice Bran | 4week bid | powder | 6/20(30%) |
| X. D. Tang, et al.2018^[32]^ | China | RomeⅢ/  IBS-D | English | Multicenter | 109/**107** | 42.4±13.96 | 66/107 | Chang’an I Recipe, 150ml | 8week tid | liquid | 43/107(40%) |
| M. A. L. Van Tilburg, et al.2014^[33]^ | America | RomeⅢ  / | English | Single center | 15/15/  **15** | / | / | Ginger, 1g/2g/day | 4week qd | capsule | 8/15(53%) |
| Lijun Cai, et al.2013^[34]^ | China | RomeⅢ/  IBS-D | Chinese | Single center | 18/**19** | 41.8± 9.33 | 4/14 | decoction of dispersing stagnated liver-qi,invigorating spleen and warming kidney, 150ml | 8week tid | liquid | 11/19 (58%) |
| Yimeng Li, et al.2010^[35]^ | China | RomeⅢ/  IBS-D | Chinese | Single center | 30/**30** | 36.6±14.49 | 14/30 | Chang Ji Tai Granule, 45g/bag | 4week bid | granules | 18/30 (60%) |
| Yingjie Si, et al.2023^[36]^ | China | RomeIV/  IBS-D | Chinese | Multicenter | 42/43/  **35** | 44.6±13.40 | 14/35 | Shuchang Decoction,12g | 8week bid | granules | 12/35(34%) |
| Bin Yu and Feng Ma2011^[37]^ | China | RomeⅢ/  All | Chinese | Single center | 20/20/  **20** | 50.9±8.93） | 9/11 | Emotional Intervention with Modified Pain-diarrhea Decoction, 100ml | 4week tid | liquid | 8/20(40％) |
| Zhengli Zhang, et al.2012^[38]^ | China | RomeⅢ/  IBS-D | Chinese | Single center | 42/**30** | / | / | Chang Ji Tai Granule | 4week bid | granules | 16/30（53%） |

**Table S1 The basic characteristics of 24 researches**

E, Experimental Group; P, Placebo Group; “/”,Lack of valid data. IBS-D: irritable bowel syndrome-diarrhea; IBS-C: irritable bowel syndrome-constipation; bid: bis in die (Latin, twice daily); tid: ter in die (Latin, three times a day); qid: quarter in die (Latin, four times a day); qd: quaque die (Latin, once a day)
