# Supplementary material for: Herbal placebo response in clinical trials on irritable bowel syndrome: a systematic review and meta-analysis
Source: Front Pharmacol. 2024 Nov 28;15:1475366. doi: 10.3389/fphar.2024.1475366 (PMC11634590; doi:10.3389/fphar.2024.1475366)
Supplement: Supplementary file 2 [file Table2.docx]

| **Table S2 the results of subgroup analysis** | | | | | |
| --- | --- | --- | --- | --- | --- |
| **subtype** | **Number** | | **size** | **Proportion 95%CI** | **P value for I^2^** |
| **diagnostic criteria** | | | | | |
| Rome I | 2 | 73 | | 0.26 (0.16; 0.37) | P=0.32 |
| Rome II | 4 | 173 | | 0.38 (0.25; 0.53) | P=0.02 |
| Rome Ⅲ | 15 | 715 | | 0.39(0.30; 0.48) | P<0.01 |
| Rome Ⅳ | 3 | 190 | | 0.35(0.28; 0.42) | P=0.96 |
| **Duration of treatment** | | | | | |
| 4 weeks | 13 | 618 | | 0.37(0.31; 0.44) | P<0.01 |
| 8weeks | 6 | 331 | | 0.43(0.36; 0.51) | P=0.11 |
| 12week | 2 | 73 | | 0.40(0.07; 0.78) | P<0.01 |
| **IBS Subtypes** | | | | | |
| IBS-C | 3 | 137 | | 0.34(0.21; 0.48) | P=0.06 |
| IBS-D | 11 | 669 | | 0.39(0.32; 0.46) | P<0.01 |
| All | 9 | 330 | | 0.34(0.22; 0.47) | P<0.01 |
| **research locations** | | | | | |
| European | 5 | 220 | | 0.30(0.14; 0.49) | P<0.01 |
| Australian | 2 | 99 | | 0.39(0.27; 0.51) | P=0.24 |
| China | 10 | 620 | | 0.41(0.37; 0.45) | P=0.17 |
| Asian(excluding China) | 5 | 162 | | 0.26(0.18; 0.36) | P=0.19 |
| American | 2 | 50 | | 0.58(0.44; 0.72) | P=0.66 |
| **forms** | | | | | |
| Capsules | 7 | 307 | | 0.31(0.17;0.47) | P<0.01 |
| granules | 6 | 254 | | 0.41(0.33; 0.50) | P=0.13 |
| syrups | 2 | 62 | | 0.30(0.17; 0.45) | P=0.22 |
| liquid | 5 | 318 | | 0.39(0.33;0.44) | P=0.47 |
| tablets | 3 | 190 | | 0.45(0.31; 0.60) | P=0.06 |
| **low concentration of herbal ingredients** | | | | | |
| contained | 4 | 202 | | 0.46(0.35; 0.56) | P<0.01 |
| Not contained | 20 | 949 | | 0.35(0.29; 0.42) | P<0.01 |
| **language** | | | | | |
| English | 19 | 1017 | | 0.35(0.28; 0.41) | P<0.01 |
| Chinese | 5 | 134 | | 0.49(0.38; 0.59) | P=0.22 |
