# Supplementary material for: Herbal placebo response in clinical trials on irritable bowel syndrome: a systematic review and meta-analysis
Source: Front Pharmacol. 2024 Nov 28;15:1475366. doi: 10.3389/fphar.2024.1475366 (PMC11634590; doi:10.3389/fphar.2024.1475366)

Supplementary

search strategy:

Pubmed:

((((((((((((((Irritable Bowel Syndrome[MeSH Terms]) OR (functional disease, colon[MeSH Terms])) OR (Irritable Bowel Syndromes[Title/Abstract])) OR (Syndrome, Irritable Bowel[Title/Abstract])) OR (Syndromes, Irritable Bowel[Title/Abstract])) OR (Colon, Irritable[Title/Abstract])) OR (Irritable Colon[Title/Abstract])) OR (Colitis, Mucous[Title/Abstract])) OR (Colitides, Mucous[Title/Abstract])) OR (Mucous Colitides[Title/Abstract])) OR (Mucous Colitis[Title/Abstract])) OR (IBS[Title/Abstract])) OR (spastic colon[Title/Abstract])) AND ((((((((((((((Traditional Chinese Medicine[MeSH Terms]) OR (Drugs, Chinese Herbal[MeSH Terms])) OR (Phytotherapy[MeSH Terms])) OR (chinese medic*[Title/Abstract])) OR (chinese herb*[Title/Abstract])) OR (chinese drug*[Title/Abstract])) OR (chinese formul*[Title/Abstract])) OR (chinese plant*[Title/Abstract])) OR (chinese prescri*[Title/Abstract])) OR (complementary therap*[Title/Abstract])) OR (alternativ* treatment*[Title/Abstract])) OR (alternativ* therap*[Title/Abstract])) OR (alternativ* medicin*[Title/Abstract])) OR (complementary therap*[Title/Abstract]))) AND (((randomized controlled trial[Publication Type]) OR (randomized[Title/Abstract])) OR (placebo[Title/Abstract]))

Cochrane:

#1 MeSH descriptor: [Irritable Bowel Syndrome] explode all trees 1642

#2 (Irritable Bowel Syndromes):ti,ab,kw OR (Syndrome, Irritable Bowel):ti,ab,kw OR (Syndromes, Irritable Bowel):ti,ab,kw OR (Colon, Irritable):ti,ab,kw OR (Irritable Colon):ti,ab,kw (Word variations have been searched) 4992

#3 (Colitis, Mucous):ti,ab,kw OR (Colitides, Mucous):ti,ab,kw OR (Mucous Colitides):ti,ab,kw OR (Mucous Colitis):ti,ab,kw OR (IBS):ti,ab,kw (Word variations have been searched) 7358

#4 (spastic colon):ti,ab,kw (Word variations have been searched) 26

#5 #1 OR #2 OR #3 OR #4 9150

#6 MeSH descriptor: [Medicine, Chinese Traditional] explode all trees 1568

#7 MeSH descriptor: [Drugs, Chinese Herbal] explode all trees 4197

#8 MeSH descriptor: [Phytotherapy] explode all trees 4762

#9 (chinese medic*):ti,ab,kw OR (chinese herb*):ti,ab,kw OR (chinese drug*):ti,ab,kw OR (chinese formul*):ti,ab,kw OR (chinese plant*):ti,ab,kw (Word variations have been searched) 28139

#10 (chinese prescri*):ti,ab,kw OR (complementary therap*):ti,ab,kw OR (alternativ* treatment*):ti,ab,kw OR (alternativ* therap*):ti,ab,kw (Word variations have been searched) 49469

#11 (alternativ* medicin*):ti,ab,kw OR (complementary therap*):ti,ab,kw (Word variations have been searched) 10174

#12 #6 OR #7 OR #8 OR #9 OR #10 OR #11 79194

#13 #5 AND #12 569

Embase:

No. Query Results	Results	Date
#36. #13 AND #28 AND #35	459	12 Nov 2023
#35. #29 OR #30 OR #31 OR #32 OR #33 OR #34	1,758,275	12 Nov 2023
#34. 'randomly':ab,ti,kw	557,978	12 Nov 2023
#33. 'randomised':ab,ti,kw	195,541	12 Nov 2023

**#32. 'placebo':ab,ti,kw**	**368,728**	**12 Nov 2023**
**#31. 'randomized':ab,ti,kw**	**978,344**	**12 Nov 2023**
**#30. 'double-blind':ti,kw**	**64,722**	**12 Nov 2023**
**#29. 'random':ti,kw**	**34,222**	**12 Nov 2023**
**#28. #14 OR #15 OR #16 OR #17 OR #18 OR #19 OR #20 OR #21 OR #22 OR #23 OR #24 OR #25 OR #26 OR #27**	**395,666**	**12 Nov 2023**
**#27. 'complementary therap*':ab,ti,kw**	**7,868**	**12 Nov 2023**
**#26. 'alternativ* medicin*':ab,ti,kw**	**18,631**	**12 Nov 2023**
**#25. 'alternativ* therap*':ab,ti,kw**	**26,171**	**12 Nov 2023**
**#24. 'alternativ* treatment*':ab,ti,kw**	**34,766**	**12 Nov 2023**
**#23. 'complementary therap*':ab,ti,kw**	**7,868**	**12 Nov 2023**
**#22. 'chinese prescri*':ab,ti,kw**	**352**	**12 Nov 2023**
**#21. 'chinese plant*':ab,ti,kw**	**189**	**12 Nov 2023**
**#20. 'chinese formul*':ab,ti,kw**	**470**	**12 Nov 2023**
**#19. 'chinese drug':ab,ti,kw**	**665**	**12 Nov 2023**
**#18. 'chinese medic*':ab,ti,kw**	**70,585**	**12 Nov 2023**
**#17. 'chinese medic*':ab,ti,kw**	**70,585**	**12 Nov 2023**
**#16. 'phytotherapy'/exp OR 'phytotherapy'**	**30,225**	**12 Nov 2023**
**#15. 'herbaceous agent'/exp**	**60,984**	**12 Nov 2023**
**#14. 'chinese medicine'/exp OR 'chinese medicine'**	**241,783**	**12 Nov 2023**
**#13. #1 OR #2 OR #3 OR #4 OR #5 OR #6 OR #7 OR #8 OR #9 OR #10 OR #11 OR #12**	**37,839**	**12 Nov 2023**
**#12. 'spastic colon':ab,ti,kw**	**101**	**12 Nov 2023**
**#11. 'ibs':ab,ti,kw**	**20,691**	**12 Nov 2023**
**#10. 'mucous colitis':ab,ti,kw**	**18**	**12 Nov 2023**
**#9. 'mucous colitides':ab,ti,kw**	**1**	**12 Nov 2023**
**#8. 'colitides, mucous':ab,ti,kw**	**1**	**12 Nov 2023**
**#7. 'colitis, mucous':ab,ti,kw**	**10**	**12 Nov 2023**
**#6. 'irritable colon':ab,ti,kw**	**595**	**12 Nov 2023**
**#5. 'colon, irritable':ab,ti,kw**	**21**	**12 Nov 2023**
**#4. 'syndromes, irritable bowel':ab,ti,kw**	**10**	**12 Nov 2023**
**#3. 'syndrome, irritable bowel':ab,ti,kw**	**89**	**12 Nov 2023**
**#2. 'irritable bowel syndromes':ab,ti,kw**	**51**	**12 Nov 2023**
**#1. 'irritable colon'/exp OR 'irritable colon'**	**32,851**	**12 Nov 2023**

CNKI (Chinese database with search terms in Chinese)

(主题:肠易激综合征)**OR**(篇关摘:腹泻型肠易激综合征 + 便秘型肠易激综合征 + 过敏性结肠炎 + 黏液性结肠炎 + **IBS**(精确))**AND**(主题:中医药 + 草本剂 + 植物治疗法)**OR**(篇关摘:中医* + 中药* + 中药汤剂 + 中国植物 + 中药处方 + 补充治疗 + 替代治疗 + 补充医学 + 替代医学 + 中草药 + 植物药 + 药用植物 + 植物提取物 + 植物制剂 + 植物，药用(精确))**AND**(主题:随机对照试验)**OR**(篇关摘:随机 + 安慰剂(精确))，过滤器：会议论文、毕业论文、成果

WanFang (Chinese database with search terms in Chinese)

(((((主题=(肠易激综合征 or 腹泻型肠易激综合征 or 便秘型肠易激综合征 or 过敏性结肠炎 or 黏液性结肠炎 or IBS)) AND (主题=中医药 or 草本剂 or 植物治疗法 or 中医* or 中药* or 中药汤剂 or 中国植物 or 中药处方 or 补充治疗 or 替代治疗 or 补充医学 or 替代医学 or 中草药 or 植物药 or 药用植物 or 植物提取物)) AND (主题=随机对照试验 or 随机 or 安慰剂)))) AND ((安慰剂) OR 安慰剂组)

Sinomed (Chinese database with search terms in Chinese)

(("安慰剂"[全部字段:智能]) AND ("随机对照试验"[常用字段:智能] OR "随机"[常用字段:智能] OR "安慰剂"[常用字段:智能]) AND (("中医药"[常用字段:智能] OR "草本剂"[常用字段:智能] OR "植物治疗法"[常用字段:智能] OR "中医*"[常用字段:智能] OR "中药*"[常用字段:智能] OR "中药汤剂"[常用字段:智能] OR "中国植物"[常用字段:智能] OR "中药处方"[常用字段:智能] OR "补充治疗"[常用字段:智能]) OR ("中医药"[常用字段:智能] OR "草本剂"[常用字段:智能] OR "植物治疗法"[常用字段:智能] OR "中医*"[常用字段:智能] OR "中医*"[常用字段:智能] AND "or"[常用字段:智能] AND "中药*"[常用字段:智能] AND "or"[常用字段:智能] AND "中药汤剂"[常用字段:智能])) AND ("肠易激综合征"[常用字段:智能] OR "腹泻型肠易激综合征"[常用字段:智能] OR "便秘型肠易激综合征"[常用字段:智能] OR "过敏性结肠炎"[常用字段:智能] OR "黏液性结肠炎"[常用字段:智能] OR "IBS"[常用字段:智能]))

Table S1 Assessment for risk of bias

Study	Random	Allocation	Blinding patients	Blinding researcher	Incomplete	Selective
F. Alt, et al. 2017	low	low	low	low	high	low
A. Bensoussan, et al. 2015	low	low	low	low	high	low
A. Bensoussan, et al. 1998	high	low	low	low	high	low
M. Chen, et al. 2018	low	low	low	low	high	low
K. Davis, et al. 2006	low	low	low	low	high	low
P. Heydari, et al. 2023	low	unclear	unclear	unclear	high	low
Y. Lai, et al. 2022	low	low	low	low	high	low
J. H. Lee, et al. 2019	low	high	low	high	high	low
W. K. Leung, et al. 2006	low	low	low	low	high	unclear
A. Madisch, et al. 2004	low	low	low	low	low	low
H. K. Pazhouh, et al. 2020	low	low	low	low	high	unclear
P. Portincasa, et al. 2016	low	unclear	unclear	unclear	high	low
Y. A. Saito, et al. 2010	low	low	low	low	high	high
S. Sallon, et al. 2002	low	unclear	unclear	low	high	low
S. St?rsrud, et al. 2015	low	high	low	low	high	low
X. Su, et al. 2013	low	unclear	unclear	unclear	high	unclear
K. Takeshi, et al. 2014	low	unclear	unclear	unclear	low	low
X. D. Tang, et al. 2018	low	low	low	low	high	low
M. A. L. Van Tilburg, et al. 2014	unclear	unclear	unclear	unclear	high	unclear
L.J. Cai, et al. 2013	low	low	low	low	high	low
L.M. Li, et al. 2010	low	low	low	low	high	low
Y.J. Si, et al.2023	low	unclear	low	low	high	low
B. Yu, et al.2011	unclear	unclear	unclear	unclear	unclear	low
Z.L. Zhang, et al. 2012	unclear	unclear	unclear	unclear	unclear	unclear

Table S2 Assessment for risk of the 4 excluded dissertations

Study	Random	allocation	Blinding patients	Blinding researcher	Incomplete	Selective
S.G. Huang 2015	high	high	high	unclear	unclear	unclear
Y.Niu 2016	low	low	low	low	high	high
Y.L. Wu 2021	low	low	low	low	high	high
X.M. Xu 2022	low	unclear	unclear	unclear	high	high

Table S3 The result of meta-regression analysis

Subgroup analysis	P-val
Criteria	0.6297
Location	*0.0438
Endpoint	*0.0365
Type	0.5946
Contains Chinese medicine	0.1845
IBS Subtype	0.6920
Language	0.0629

Figure S1 Effect of diagnostic criteria on response rate

Study or Subgroup	Events	Total	IV, Random, 95% CI	Weight
**Rome Ⅲ**				
F. Alt, et al. 2017	27	47	0.57 [0.42; 0.72]	7.2%
A. Bensoussan, et al. 2015	28	64	0.44 [0.31; 0.57]	7.7%
M. Chen, et al. 2018	30	80	0.38 [0.27; 0.49]	8.0%
P. Heydari, et al. 2023	8	49	0.16 [0.08; 0.30]	7.3%
J. H. Lee, et al. 2019	7	20	0.35 [0.16; 0.59]	5.4%
P. Portincasa, et al. 2016	4	59	0.07 [0.02; 0.16]	7.6%
S. St?rsrud, et al. 2015	11	35	0.31 [0.17; 0.49]	6.6%
X. Su, et al. 2013	53	120	0.44 [0.35; 0.54]	8.5%
K. Takeshi, et al. 2014	6	20	0.30 [0.13; 0.54]	5.4%
X. D. Tang, et al. 2018	43	107	0.40 [0.31; 0.50]	8.3%
M. A. L. Van Tilburg, et al. 2014	8	15	0.53 [0.27; 0.78]	4.8%
L.J. Cai, et al. 2013	11	19	0.58 [0.34; 0.79]	5.3%
Y.M. Li, et al. 2010	18	30	0.60 [0.41; 0.77]	6.3%
B. Yu, et al.2011	8	20	0.40 [0.20; 0.64]	5.4%
Z.L. Zhang, et al. 2012	16	30	0.53 [0.34; 0.72]	6.3%
**Total (95% CI)**		**715**	**0.39 [0.30; 0.48]**	**100.0%**
Heterogeneity: $Tau^2 = 0.0229$; $Chi^2 = 71.56$, df = 14 ($P < 0.01$); $I^2 = 80\%$				
**Rome Ⅰ**				
A. Bensoussan, et al. 1998	11	35	0.31 [0.17; 0.49]	49.4%
S. Sallon, et al. 2002	8	38	0.21 [0.09; 0.37]	50.6%
**Total (95% CI)**		**73**	**0.26 [0.16; 0.37]**	**100.0%**
Heterogeneity: $Tau^2 = 0$; $Chi^2 = 0.98$, df = 1 ($P = 0.32$); $I^2 = 0\%$				
**Rome Ⅱ**				
K. Davis, et al. 2006	6	27	0.22 [0.10; 0.42]	22.0%
W. K. Leung, et al. 2006	20	59	0.34 [0.21; 0.47]	27.4%
A. Madisch, et al. 2004	20	52	0.38 [0.25; 0.53]	26.6%
Y. A. Saito, et al. 2010	21	35	0.60 [0.42; 0.76]	24.0%
**Total (95% CI)**		**173**	**0.38 [0.25; 0.53]**	**100.0%**
Heterogeneity: $Tau^2 = 0.0160$; $Chi^2 = 10.04$, df = 3 ($P = 0.02$); $I^2 = 70\%$				
**Rome IV**				
Y. Lai, et al. 2022	42	120	0.35 [0.26; 0.44]	38.9%
H. K. Pazhouh, et al. 2020	13	35	0.37 [0.21; 0.55]	30.5%
Y.J. Si, et al.2023	12	35	0.34 [0.19; 0.52]	30.5%
**Total (95% CI)**		**190**	**0.35 [0.28; 0.42]**	**100.0%**
Heterogeneity: $Tau^2 = 0$; $Chi^2 = 0.08$, df = 2 ($P = 0.96$); $I^2 = 0\%$				
Heterogeneity: $Tau^2 = 0.0160$; $Chi^2 = 87.33$, df = 23 ($P < 0.01$); $I^2 = 74\%$				
Test for subgroup differences: $Chi^2 = 3.80$, df = 3 ($P = 0.28$)				

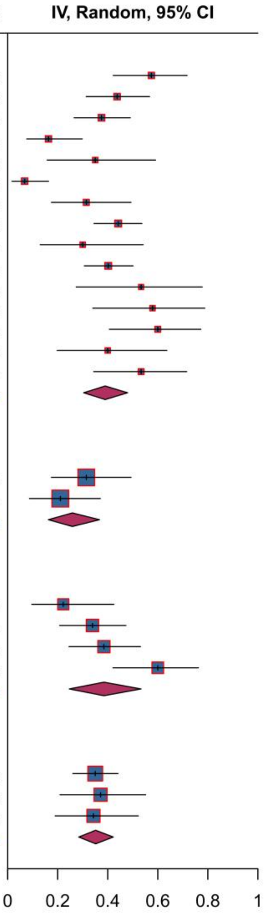

Figure S2 Effects of IBS Subtypes on Herbal Placebo Response Rates

Subgroup	Events	Total	IV, Random, 95% CI	Weight
**IBS (not subtyped)**				
F. Alt, et al. 2017	27	47	0.57 [0.42; 0.72]	12.2%
A. Bensoussan, et al. 1998	11	35	0.31 [0.17; 0.49]	11.3%
K. Davis, et al. 2006	6	27	0.22 [0.09; 0.42]	10.3%
A. Madisch, et al. 2004	20	52	0.38 [0.25; 0.53]	12.5%
P. Portincasa, et al. 2016	4	59	0.07 [0.01; 0.16]	12.8%
Y. A. Saito, et al. 2010	21	35	0.60 [0.42; 0.76]	11.3%
S. St?rsrud, et al. 2015	11	35	0.31 [0.17; 0.49]	11.3%
K. Takeshi, et al. 2014	6	20	0.30 [0.12; 0.54]	9.2%
B. Yu, et al.2011	8	20	0.40 [0.20; 0.64]	9.2%
**Total (95% CI)**		**330**	**0.34 [0.22; 0.47]**	**100.0%**
Heterogeneity: $Tau^2 = 0.0297$; $Chi^2 = 51.3$, df = 8 (P < 0.01); $I^2 = 84\%$				
**IBS−C**				
A. Bensoussan, et al. 2015	28	64	0.44 [0.31; 0.57]	36.4%
H. K. Pazhouh, et al. 2020	13	35	0.37 [0.21; 0.55]	31.4%
S. Sallon, et al. 2002	8	38	0.21 [0.10; 0.37]	32.2%
**Total (95% CI)**		**137**	**0.34 [0.21; 0.48]**	**100.0%**
Heterogeneity: $Tau^2 = 0.0098$; $Chi^2 = 5.53$, df = 2 (P = 0.06); $I^2 = 64\%$				
**IBS−D**				
M. Chen, et al. 2018	30	80	0.38 [0.27; 0.49]	10.2%
P. Heydari, et al. 2023	8	49	0.16 [0.07; 0.30]	9.3%
Y. Lai, et al. 2022	42	120	0.35 [0.26; 0.44]	10.8%
J. H. Lee, et al. 2019	7	20	0.35 [0.17; 0.59]	7.0%
W. K. Leung, et al. 2006	20	59	0.34 [0.21; 0.47]	9.7%
X. Su, et al. 2013	53	120	0.44 [0.35; 0.54]	10.8%
X. D. Tang, et al. 2018	43	107	0.40 [0.30; 0.50]	10.7%
L.J.Cai, et al. 2013	11	19	0.58 [0.34; 0.79]	6.8%
L.M. Li, et al. 2010	18	30	0.60 [0.41; 0.77]	8.1%
Y.J. Si, et al.2023	12	35	0.34 [0.19; 0.52]	8.5%
Z.L. Zhang, et al. 2012	16	30	0.53 [0.34; 0.72]	8.1%
**Total (95% CI)**		**669**	**0.39 [0.32; 0.46]**	**100.0%**
Heterogeneity: $Tau^2 = 0.0084$; $Chi^2 = 26.07$, df = 10 (P < 0.01); $I^2 = 62\%$				
Heterogeneity: $Tau^2 = 0.0161$; $Chi^2 = 85.68$, df = 22 (P < 0.01); $I^2 = 74\%$				
Test for subgroup differences: $Chi^2 = 0.73$, df = 2 (P = 0.69)				

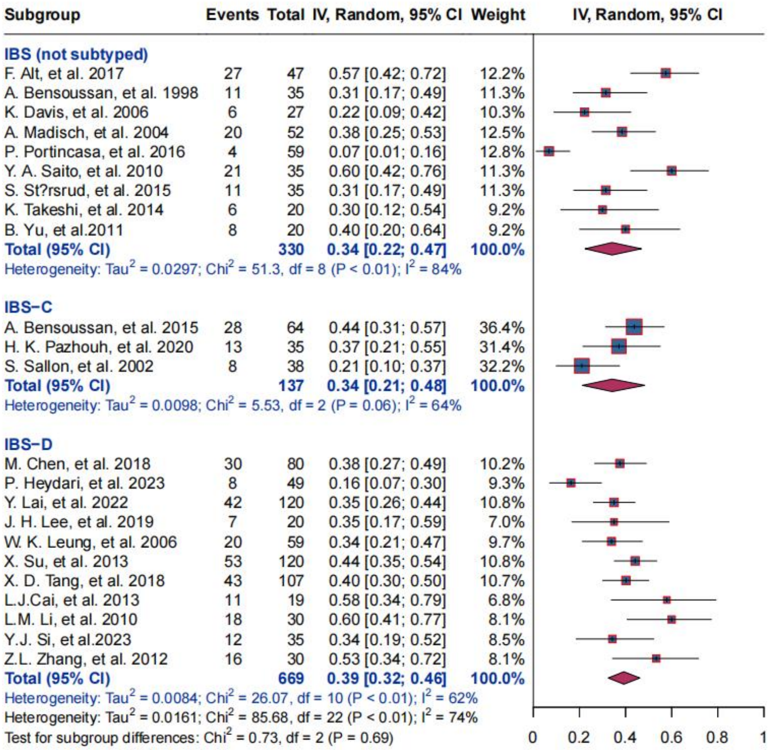

Figure S3 Effect of placebo forms on response rate

Study or Subgroup	Events	Total	IV, Random, 95% CI	Weight
**capsule**				
F. Alt.2017	27	47	0.57 [0.42; 0.72]	15.0%
A. Bensoussan. 2015	28	64	0.44 [0.31; 0.57]	16.0%
A. Bensoussan. 1998	11	35	0.31 [0.17; 0.49]	13.9%
P. Heydari. 2023	8	49	0.16 [0.08; 0.30]	15.1%
P. Portincasa. 2016	4	59	0.07 [0.01; 0.16]	15.8%
S. Sallon. 2002	8	38	0.21 [0.10; 0.37]	14.2%
M.A.L.Van Tilburg. 2014	8	15	0.53 [0.28; 0.77]	10.0%
**Total (95% CI)**		**307**	**0.31 [0.17; 0.47]**	**100.0%**
Heterogeneity: $Tau^2 = 0.0403$; $Chi^2 = 51.52$, df = 6 (P < 0.01); $I^2 = 88\%$				
**granules**				
M. Chen. 2018	30	80	0.38 [0.26; 0.49]	19.8%
J. H. Lee. 2019	7	20	0.35 [0.16; 0.59]	13.5%
W. K. Leung.2006	20	59	0.34 [0.22; 0.47]	18.7%
Li Yimeng 2010	18	30	0.60 [0.41; 0.77]	15.7%
Si Yingjie 2023	12	35	0.34 [0.19; 0.52]	16.5%
Zhang Zhengli 2012	16	30	0.53 [0.34; 0.72]	15.7%
**Total (95% CI)**		**254**	**0.41 [0.33; 0.50]**	**100.0%**
Heterogeneity: $Tau^2 = 0.0043$; $Chi^2 = 8.54$, df = 5 (P = 0.13); $I^2 = 41\%$				
**syrups**				
K. Davis. 2006	6	27	0.22 [0.09; 0.42]	47.9%
H. K. Pazhou. 2020	13	35	0.37 [0.21; 0.55]	52.1%
**Total (95% CI)**		**62**	**0.30 [0.17; 0.45]**	**100.0%**
Heterogeneity: $Tau^2 = 0.0042$; $Chi^2 = 1.52$, df = 1 (P = 0.22); $I^2 = 34\%$				
**liquid**				
Y. Lai. 2022	42	120	0.35 [0.27; 0.44]	24.1%
A. Madisch. 2004	20	52	0.38 [0.26; 0.53]	21.1%
X. D. Tang 2018	43	107	0.40 [0.30; 0.50]	23.8%
Cai Lijun 2013	11	19	0.58 [0.33; 0.80]	15.3%
Yu Bin 2011	8	20	0.40 [0.20; 0.64]	15.7%
**Total (95% CI)**		**318**	**0.39 [0.33; 0.44]**	**100.0%**
Heterogeneity: $Tau^2 = < 0.0001$; $Chi^2 = 3.58$, df = 4 (P = 0.47); $I^2 = 0\%$				
**tablet**				
Y. A. Saito. 2010	21	35	0.60 [0.42; 0.76]	30.6%
S. St.rsrud. 2015	11	35	0.31 [0.16; 0.49]	30.6%
X. Su.2013	53	120	0.44 [0.35; 0.54]	38.7%
**Total (95% CI)**		**190**	**0.45 [0.31; 0.60]**	**100.0%**
Heterogeneity: $Tau^2 = 0.0110$; $Chi^2 = 5.71$, df = 2 (P = 0.06); $I^2 = 65\%$				
Heterogeneity: $Tau^2 = 0.0167$; $Chi^2 = 86.98$, df = 22 (P < 0.01); $I^2 = 75\%$				
Test for subgroup differences: $Chi^2 = 3.35$, df = 4 (P = 0.50)				

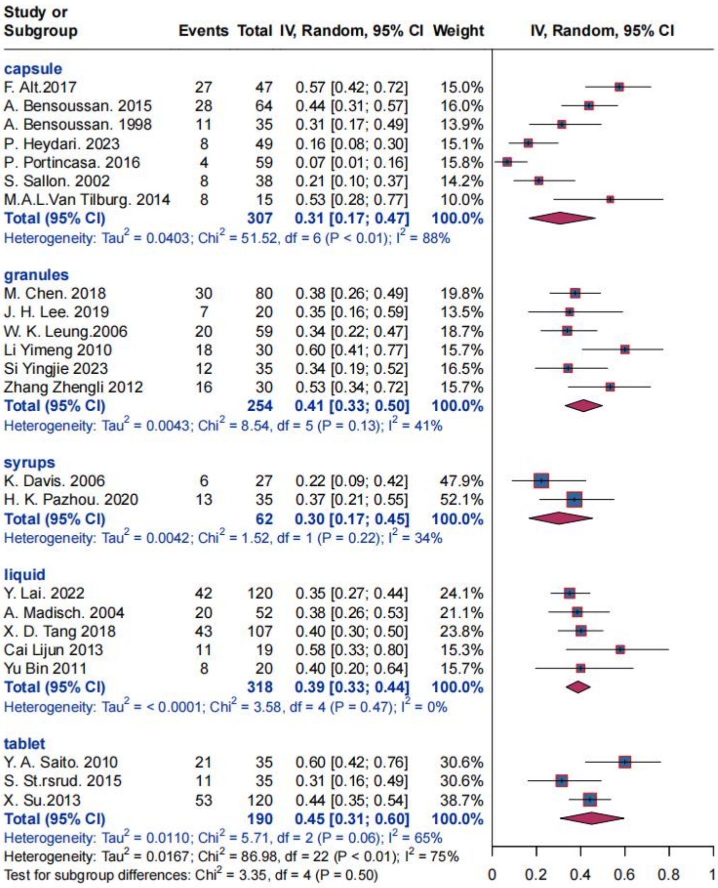

Figure S4 Effect of low concentrations of herbal ingredients on response rate

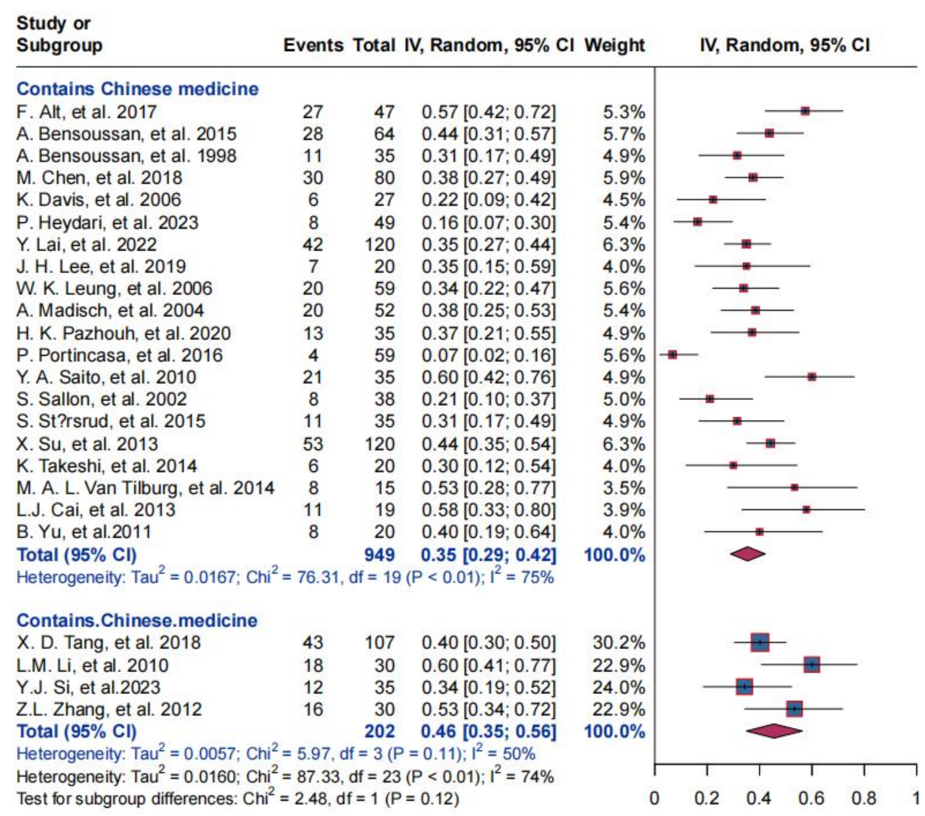

Figure S5 Effect of language on response rate

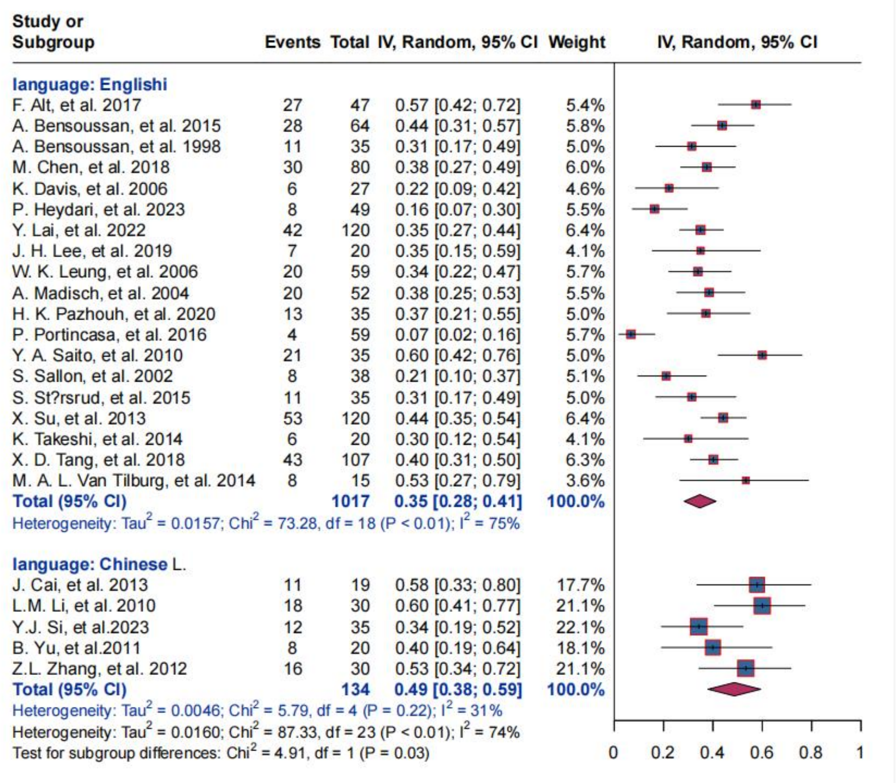

Figure S6 Effects of research locations on herbal placebo response rates

Subgroup	Events	Total	IV, Random, 95% CI	Weight
**European**				
F. Alt.2017	27	47	0.57 [0.42; 0.72]	20.6%
K. Davis. 2006	6	27	0.22 [0.09; 0.42]	17.5%
A. Madisch. 2004	20	52	0.38 [0.25; 0.53]	21.1%
P. Portincasa. 2016	4	59	0.07 [0.02; 0.17]	21.7%
S. St.rsrud. 2015	11	35	0.31 [0.17; 0.49]	19.0%
**Total (95% CI)**		**220**	**0.30 [0.14; 0.49]**	**100.0%**
Heterogeneity: $Tau^2 = 0.0424$; $Chi^2 = 39.03$, df = 4 ($P < 0.01$); $I^2 = 90\%$				
**Australia**				
A. Bensoussan. 2015	28	64	0.44 [0.31; 0.57]	53.7%
A. Bensoussan. 1998	11	35	0.31 [0.17; 0.49]	46.3%
**Total (95% CI)**		**99**	**0.39 [0.27; 0.51]**	**100.0%**
Heterogeneity: $Tau^2 = 0.0021$; $Chi^2 = 1.39$, df = 1 ($P = 0.24$); $I^2 = 28\%$				
**Asian**				
M. Chen. 2018	30	80	0.38 [0.26; 0.49]	7.8%
P. Heydari. 2023	8	49	0.16 [0.07; 0.30]	7.1%
Y. Lai. 2022	42	120	0.35 [0.26; 0.44]	8.3%
J. H. Lee. 2019	7	20	0.35 [0.16; 0.59]	5.3%
W. K. Leung.2006	20	59	0.34 [0.22; 0.47]	7.4%
H. K. Pazhou. 2020	13	35	0.37 [0.21; 0.55]	6.5%
S. Sallon. 2002	8	38	0.21 [0.10; 0.37]	6.6%
X. Su.2013	53	120	0.44 [0.35; 0.54]	8.3%
K. Takeshi. 2014	6	20	0.30 [0.13; 0.54]	5.3%
X. D. Tang 2018	43	107	0.40 [0.30; 0.50]	8.2%
Cai Lijun 2013	11	19	0.58 [0.34; 0.79]	5.2%
Li Yimeng 2010	18	30	0.60 [0.40; 0.78]	6.2%
Si Yingjie 2023	12	35	0.34 [0.18; 0.52]	6.5%
Yu Bin 2011	8	20	0.40 [0.20; 0.64]	5.3%
Zhang Zhengli 2012	16	30	0.53 [0.34; 0.72]	6.2%
**Total (95% CI)**		**782**	**0.37 [0.32; 0.43]**	**100.0%**
Heterogeneity: $Tau^2 = 0.0071$; $Chi^2 = 31.64$, df = 14 ($P < 0.01$); $I^2 = 56\%$				
**America**				
Y. A. Saito. 2010	21	35	0.60 [0.42; 0.77]	58.2%
M.A.L.Van Tilburg. 2014	8	15	0.53 [0.27; 0.78]	41.8%
**Total (95% CI)**		**50**	**0.58 [0.44; 0.72]**	**100.0%**
Heterogeneity: $Tau^2 = 0$; $Chi^2 = 0.19$, df = 1 ($P = 0.66$); $I^2 = 0\%$				
Heterogeneity: $Tau^2 = 0.0160$; $Chi^2 = 87.33$, df = 23 ($P < 0.01$); $I^2 = 74\%$				
Test for subgroup differences: $Chi^2 = 8.21$, df = 3 ($P = 0.04$)				

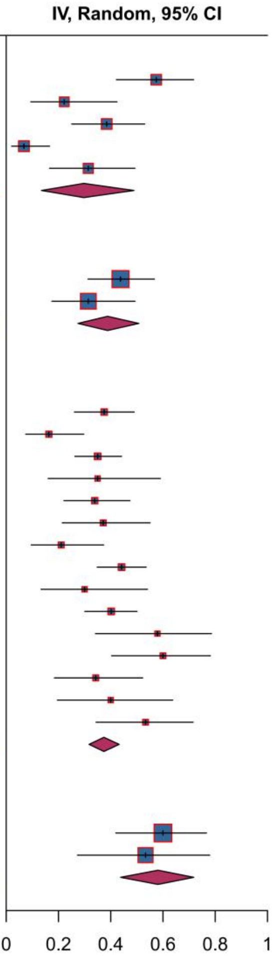

Supplement: Supplementary file 3 [file DataSheet1.pdf]
